# Revealing the Singlet Fission Mechanism for a Silane-Bridged Thienotetracene Dimer

**DOI:** 10.1021/acs.jpca.4c01463

**Published:** 2024-05-08

**Authors:** Liang-Chun Lin, Ryan D. Dill, Karl J. Thorley, Sean R. Parkin, John E. Anthony, Justin C. Johnson, Niels H. Damrauer

**Affiliations:** † Department of Chemistry, University of Colorado Boulder, Boulder, Colorado 80309, United States; ‡ Department of Chemistry & Center for Applied Energy Research, University of Kentucky, Lexington, Kentucky 40506-0055, United States; § National Renewable Energy Laboratory, 15013 Denver West Parkway, Golden, Colorado 80401, United States; ∥ Renewable and Sustainable Energy Institute (RASEI), University of Colorado Boulder, Boulder, Colorado 80309, United States

## Abstract

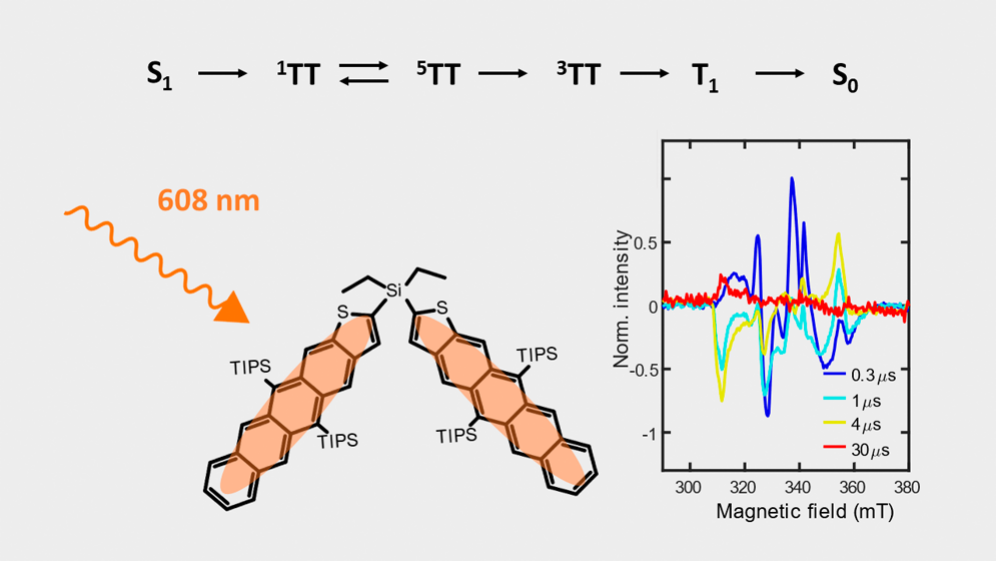

Tetraceno[2,3-*b*]thiophene is regarded as a strong candidate for singlet fission-based solar cell applications due to its mixed characteristics of tetracene and pentacene that balance exothermicity and triplet energy. An electronically weakly coupled tetraceno[2,3-*b*]thiophene dimer (Et_2_Si(TIPSTT)_2_) with a single silicon atom bridge has been synthesized, providing a new platform to investigate the singlet fission mechanism involving the two acene chromophores. We study the excited state dynamics of Et_2_Si(TIPSTT)_2_ by monitoring the evolution of multiexciton coupled triplet states, ^1^TT to ^5^TT to ^3^TT to T_1_ + S_0_, upon photoexcitation with transient absorption, temperature-dependent transient absorption, and transient/pulsed electron paramagnetic resonance spectroscopies. We find that the photoexcited singlet lifetime is 107 ps, with 90% evolving to form the TT state, and the complicated evolution between the multiexciton states is unraveled, which can be an important reference for future efforts toward tetraceno[2,3-*b*]thiophene-based singlet fission solar cells.

## Introduction

Singlet fission (SF) is a process that generates two excitons upon single photon excitation with participation of two molecular chromophores. Materials can go through SF if the energetic criterion E(S_1_) ≥ 2E(T_1_) is satisfied. The doubling of the exciton yield from one photon could push the solar cell efficiency beyond the Shockley–Queisser limit^1,2^ if coupled with a complementary second layer that efficiently produces one exciton per photon. SF application toward solar cell efficiency enhancement has been widely discussed and researched, including the SF mechanism, as it varies for chromophores in thin-film and organic dimer systems.^3−6^ Among the chromophores, tetracene- and pentacene-derived molecules are extensively investigated due to their favorable energetics between S_1_ and the coupled triplet pair, ^1^TT. However, although the large exothermicity between S_1_ and ^1^TT, ∼0.3 eV, favors SF for two pentacenes, the low triplet energy, for example, 0.8 eV in TIPS-pentacene chromophores,^7^ could make the exciton harvesting inefficient for SF-based solar cell applications. The triplet energy for tetracene, 1.25 eV,^8^ exceeds the silicon band gap, 1.1 eV, yet the small energy gap between S_1_ and ^1^TT decreases the SF efficiency.^3,9^ Here, we have studied a dimer derived from a thiophene-fused heteroacene,^10−12^ tetracene (tetraceno[2,3-*b*]thiophene or thienotetracene), that has electronic properties lying between tetracene and pentacene.^13,14^ As we will show, the thienotetracene possesses a triplet energy higher than pentacene and more favorable energetics for SF than tetracene, making it an interesting material candidate for exploiting SF in solar cell applications.

In this work, we focus on developing an understanding of the excited state dynamics, where the formation and evolution of the coupled triplet states are of importance. Considering angular momentum coupling between the two triplets on each chromophore via the exchange interaction, nine possible TT spin states are possible, including ^1^TT, ^3^TT, and ^5^TT and their substates defined by spin projection. The proposed mechanisms for multiexciton dynamics in molecular dimer systems can be generally categorized into two groups based on the spin coupling strength between the two chromophores. For strongly coupled chromophores,^15−19^^1^TT is formed from S_1_, and in the dominant loss pathway, ^1^TT or the equilibrium state of S_1_ and ^1^TT decays to ground state, S_0_ (S_1_ ⇌ ^1^TT → S_0_). For weakly coupled chromophores or when coupled triplets become weakly coupled due to triplet hopping,^14−16,20−28^^1^TT is formed from S_1_ and then forms a mixed state with ^5^TT through singlet-quintet (SQ) mixing. These efforts seek to characterize the SF mechanism in a new covalent dimer system, Et_2_Si(TIPSTT)_2_, where two thienotetracenes are connected by a silicon atom bridge.

## Results

### Et_2_Si(TIPSTT)_2_ Synthesis

**Figure 1:**
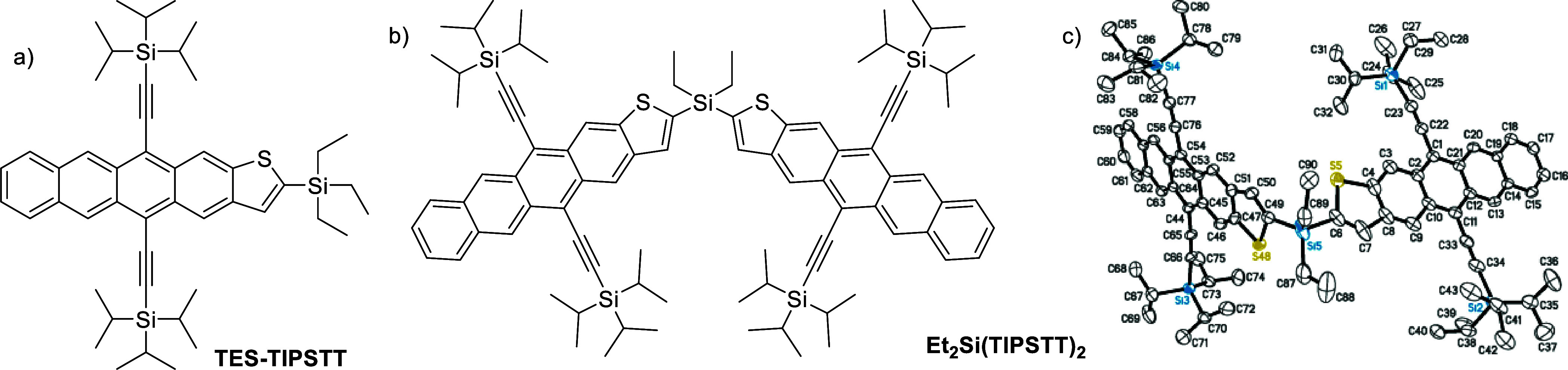
(a) Molecular structure of TES-TIPSTT (**mono**) and (b) Et_2_Si(TIPSTT)_2_ (**dimer**). (c) Ellipsoid plot of Et_2_Si(TIPSTT)_2._

The synthesis of monomeric thienotetracene TES-TIPSTT, whose structure is shown in Figure 1a, was recently described.^13^ Addition of 1 equiv of lithium diisopropylamide to bis(tri-isopropylsilyl)ethynyl thienotetracene (TIPS-TT^12^) at −78 °C resulted in deprotonation at the 2-position of the thiophene ring, and this anion could be trapped by addition of triethylchlorosilane to yield TES-TIPSTT. By analogous reaction conditions, addition of a dialkyldichlorosilane to the TIPSTT anion was used to form the dimeric Et_2_Si(TIPSTT)_2_. While this simple, scalable, 1-step reaction sequence starting from an easily prepared thienotetracene produces the desired dimer in modest (∼20%) yield, the main byproduct is the TIPSTT starting material, which could be recovered and reused. Further, the overall yield is comparable to the multistep yield of similar reported acenothiophene dimers,^29^ and the absence of any reaction step requiring transition metal catalysts eliminates any concern of contamination with heavy elements. To obtain the highest levels of material purity, chromatography of the dimer was performed both on traditional silica gel and size-exclusion stationary phases. Single crystals suitable for X-ray diffraction analysis were then grown from slow cooling of a concentrated 2-butanone solution. The crystal structure exhibits four possible positions for the sulfur atoms of the thiophene rings, indicating a possible four different conformers in the solid state. The ratios of the major:minor occupancies are 76:24 and 53:47 for the two thienotetracenes, respectively. The angle between the two acene cores, bridged by the silicon atom, is 106°, while the S–C–Si–C dihedrals differ for the two thienotetracene units, being 52° or 83°. A major intermolecular packing motif consists of a π–π interaction between thienotetrace units as well as the ethyl chains on the central silicon atom (red thienotetracenes in Figure S5). The other thienotetracene in the covalent dimer interacts with its neighbors through the TIPS groups interacting with the π surface (blue thienotetracenes in Figure S5). These different interactions explain the different geometries observed for the two thienotetracene units in the crystal structure with respect to the central silyl group. In the absence of these intermolecular interactions, it is expected that a range of thermally accessible rotational isomers are present in solution.

### Static Absorption

**Figure 2:**
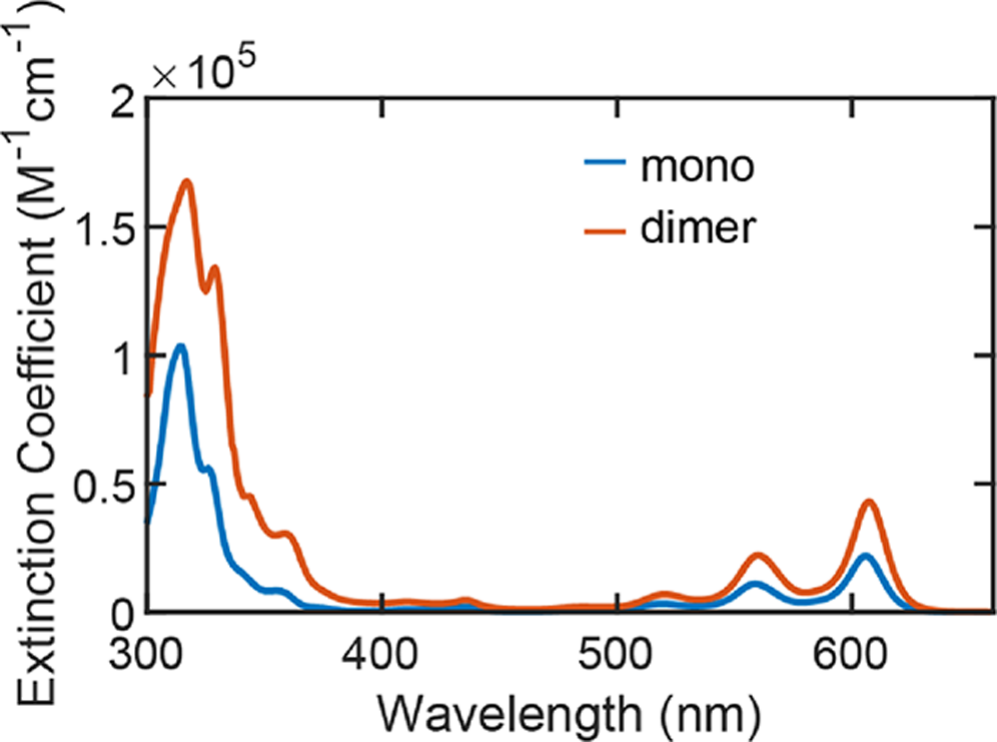
Molar extinction coefficient spectra for **mono** and **dimer** measured in toluene at room temperature.

To simplify the notation of the molecules, from here, we use **mono** to represent TES-TIPSTT and **dimer** to represent Et_2_Si(TIPSTT)_2_. A molar extinction coefficient spectrum for **mono** in room-temperature toluene is shown in Figure 2. Like other acenes in solution, it shows a red vibronic progression (∼1400 cm^–1^ spacing)—here peaked at 606 nm—corresponding to the S_0_ short axis transition. Figure S12 demonstrates the similarity between thienotetracene, tetracene, and pentacene for the HOMO and LUMO, analyzed with density functional theory. A higher energy intense absorption occurs at 315 nm and corresponds to a long-axis transition that is electronically related to S_0_ observed in unsubstituted acenes.^9^ Putting this system in some context compared to other singlet fission workhorse chromophores, S_0_ in **mono** is red-shifted relative to TIPS-tetracene (530 nm) by ∼75 nm^17^ due to participation by the fused thiophene in the delocalization of the acene. On the other hand, it is blue-shifted by 32 nm relative to TIPS-pentacene (638 nm),^17^ indicating that the thiophene increment on a tetracene core has a smaller impact than addition of a single benzene unit.^10^ These shifts are reproduced in calculations of the S_1_ energy shown in Table S1, where the T_1_ energies are also predicted. Whereas these are not calculations of dimers and there is some uncertainty, we consider the situation for **dimer** to be nearly isoergic SF (E(S_1_) ∼ 2 × E(T_1_)) in between that of TIPS-tetracene and TIPS-pentacene dimers.

From **mono** to **dimer**, an approximate doubling in extinction is observed through the visible spectral region, consistent with the 2-fold increase of chromophores per molecule. This doubling of absorption strength and the similarity in spectral shapes between **mono** and **dimer** are typical hallmarks of weak electronic coupling between chromophores. No Davydov splitting of absorption bands (Δ*E*_DV_) is observed in **dimer**, which is likely not a consequence of cancellation of opposing parallel transition dipole moments as is the case in a number of rigid systems studied in our group.^9,17^ Rather, other origins are anticipated including a distribution of Δ*E*_DV_ for thermally accessible conformers as well as weaker electronic interactions. Because toluene does not reliably form glassy matrices when it freezes, we prefer 2-methyltetrahydrofuran (MeTHF) as a spectroscopic solvent. Figure S9 demonstrates that the shape of the absorption spectrum is largely unaffected by this choice. Unless otherwise stated, data in the rest of this paper are collected with MeTHF as the solvent.

### Photoluminescence

To quantify S_1_ energies, static photoluminescence spectroscopy is utilized. As shown in Figures S10 and S11, the **mono** emission spectrum is typical of a larger acene. The vibronic progression mirrors that of the absorption spectrum and shows a small Stokes’ shift, indicating that fluorescence is from the lowest electronic excited state, S_1_. Taking the average of the absorption and emission 0–0 vibronic peaks (room-temperature MeTHF) gives an S_1_ value = 2.053 eV. Similar values are found using toluene and chloroform spectra (S_1_ = 2.043 and 2.040 eV, respectively; see the Supporting Information). These values are intermediate between TIPS-tetracene (2.31 eV; toluene) and TIPS-pentacene (1.93 eV; toluene).^17^ The **dimer** fluorescence quantum yield is dramatically lower than that of **mono** (10% versus a value for **mono** that is approaching 100%), indicating enhancement of nonradiative relaxation pathways. This is a common observation in SF systems, where spin-allowed ^1^TT formation may compete with fluorescence, although the observation of diminished emission efficiency alone is insufficient to make such an assignment.

### Time-Resolved Photoluminescence

S_1_ relaxation for both **mono** and **dimer** in room-temperature MeTHF was monitored with time-correlated single photon counting (TCSPC). The fluorescence decay of **mono** is well described by a single exponential with a 20.4 ± 0.02 ns time constant as seen in Figure S22c,d. Figure S14 shows **dimer** fluorescence decay on two timescales; the first is instrument response-limited (107 ps) and is discussed further below in the context of ultrafast measurements. The second is described by an 18.9 ± 0.02 ns time constant, similar to the relaxation of the S_1_ state of **mono**. Both relaxation timescales are associated with the same spectrum (see Figure S14b), and the fitting details are described in the Supporting Information. The similarity in fluorescence lifetimes between **mono** and **dimer** argues against delayed fluorescence as the explanation for the 18.9 ns decay of **dimer**. Delayed fluorescence lifetimes were reported for two tetracene dimers, TIPS-BT1 and TIPS-BT1′, and they show extensions to 24.3 and 36 ns, respectively, compared to an observed lifetime of 12.5 ns for monomer TIPS-Tc.^17^ One possibility for **dimer**'s 18.9 ns decay is the presence of a monomer-like impurity, but we do not believe this to be the case based on NMR and mass spectroscopy characterization. Another possible explanation is that conformational variability distorts the thermodynamics or kinetics enough to prevent SF for some subset of **dimers**.^30^

This latter explanation can follow from the **dimer** composition itself. A key feature is that there is lateral asymmetry in each chromophore arm based on which side of the thienotetracene the sulfur atom sits. Given single bond attachment of these chromophores to the central silane bridge, multiple dimer conformers are expected to exist in any sample, for example, with both sulfur atoms pointed up or down or a mixture of the two with respect to two ethyl groups on the silicon atom bridge. Notably, the single crystal diffraction data shown in Figures S4–S7 indicate a distribution of sulfur placements, suggesting that these conformers exist and are similar in energy. A cursory exploration, using density functional theory applied to a simpler model with SiH_3_ in place of TIPS, identifies three structural minima with energies falling within ∼2*k*_B_*T* (gas-phase/ground-state structures; Figure S13 and Table S2). In addition, the bulky TIPS groups on both chromophores could hinder the interconversion between the conformers. We therefore think that it is plausible that one of these conformations is responsible for the monomer-like emission, although we cannot at this time identify which one.

### Ultrafast ^1^TT Formation

**Figure 3:**
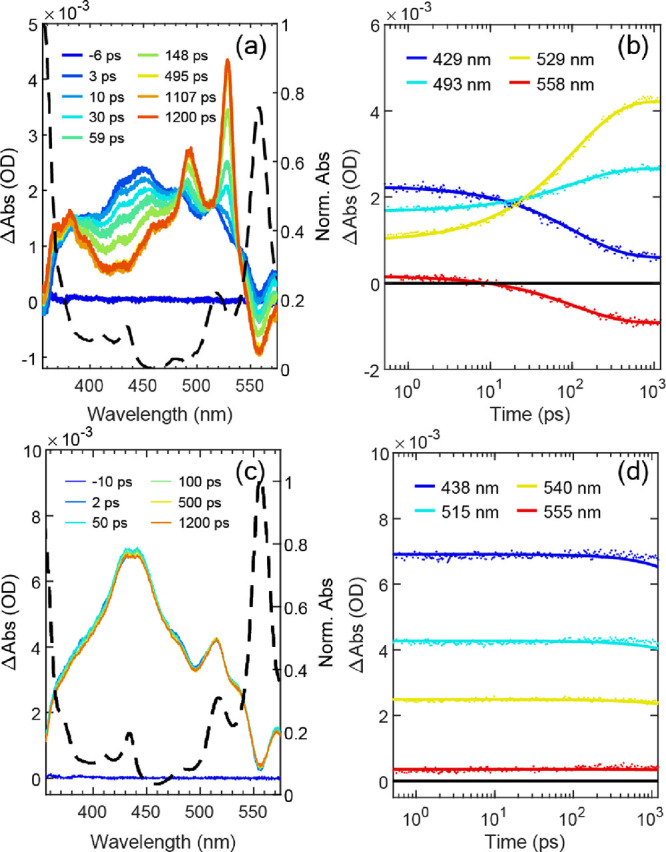
(a) fs-TA spectra of **dimer**. The excitation wavelength is at a center wavelength of 604 nm, which corresponds to S_0_, 0–0 transition. The normalized steady state UV–vis spectrum of **dimer** in MeTHF at room temperature is overlaid in a dashed black line. (b) Selected single wavelength decay time traces (dots) for **dimer** with the solid lines from the fit. (c) fs-TA spectra of **mono**. The excitation is at a center wavelength of 604 nm, which corresponds to the S_0_, 0–0 transition. The normalized steady state UV–vis spectrum of **mono** in MeTHF at room temperature is overlaid in a dashed black line. (d) Selected single wavelength decay time traces (dots) for **mono** with the solid lines from the fit.

Early time transient absorption (TA) data—from 1 ps to 1.2 ns—were collected for both **mono** and **dimer**, following ∼50 fs pulsed excitation centered at 604 nm into the 0–0 band of the respective S_1_. As seen in Figure 3c for **mono** in MeTHF at room temperature, broad excited state absorption (ESA) is observed over the spectral region interrogated (∼350–580 nm). No spectral evolution is shown, and the ESA decay lifetime is beyond the available range for the delay stage. Considering the high emission quantum yield for **mono** (*vide supra*), ESA is from the S_1_ state. Spectral slices for **dimer** in MeTHF at room temperature, selected from the full time/wavelength-resolved data set, are shown in Figure 3a. At early times, broad ESA is observed from 356 to 570 nm with an overlaid structure attributed to ground-state bleach features. Stronger bleach corresponding to the lowest energy 0–1 in the ground-state absorption spectrum gives rise to the spectral hole at 558 nm. The overall shape/quality of the early TA spectrum is consistent with what is observed for **mono** and is assigned to S_1_. Given the chromophore structure, it is not surprising that these S_1_ ESA features bear similarities to monomers or dimers utilizing TIPS-tetracene or TIPS-pentacene moieties.^7,9,17,31−33^

Subsequently, the S_1_ spectrum evolves in time with its most dramatic change, occurring on a 100 ps timescale, associated with isosbestic points at 384, 485, 513, and 539 nm. These highlight a conversion to a new species (or multiple species that are electronically similar; see below). Notably, the ESA spectrum that emerges, with a sharp band peaking at 528 nm and a corresponding bluer band at 493 nm, also bears strong resemblance to the single vibronic pattern generated at early times in TIPS-pentacene dimers that undergo the first multiexciton steps of singlet fission.^17^ The initial ∼100 ps dynamics are therefore attributed to formation of ^1^TT from S_1_. A triplet sensitization experiment (see Figure S28) confirms that this vibronic pattern is of triplet character. Although these ps TA data do not rule out intersystem crossing (ISC) in their own right, the relative speed of the process, and the resemblance of the dynamics to data collected for a number of SF active dimers built from TIPS-pentacene moieties, strongly suggests that ^1^TT is being formed as opposed to T_1_. We will show longer-time TA data that support this. A recent literature report on a related tetracenethiophene derivative also reports no significant ISC.^29^ The ∼100 ps timescale for ^1^TT formation corresponds to the fast dynamics observed using TCSPC as discussed above. Although overlapping ESA and bleach spectra prohibit deriving a quantitative TT yield from TA alone, we can reasonably take the 90% emission quenching as an estimate (with 100% being complete conversion). The small residual S_1_ ESA at long delay times is consistent with this value. We note that 2 × E(T_1_) roughly equal to E(S_1_) from the calculations might suggest that equilibrium could form and reduce the TT yield more significantly, as in some tetracene dimers.^9,17^ However, the high TT yield and the direct conversion from S_1_ to ^1^TT suggest that the process is thermodynamically driven.

Given the emergence of a sharp vibronic pattern, we initially sought to explain the data using a global model of **A** → **B**, with the assumption that **A** corresponds to S_1_ and **B** corresponds to ^1^TT prior to further evolution on timescales outside the 1.2 ns range of this experiment. In a semiquantitative sense, this model does a reasonable job representing the data leading to observation of a time constant of 94.3 ± 0.5 ps. However, there are clear residuals, suggesting that model refinement is warranted (Figure S16c). A high-quality representation of the data can be achieved using a three-component model **A** → **B** → **C**. With this model, two time constants of 21.5 ± 0.7 and 155.1 ± 2.1 ps are estimated (Figure S17c). In principle, such time constant values could be reasonable, with the shorter 21.5 ps pointing toward vibrational relaxation in the S_1_ manifold prior to multiexciton formation in 155.1 ps. With this in mind, species-associated spectra (SAS) have been analyzed and are shown in Figure S17d. While SAS for species **C** does have spectral features that would coincide with expectations for ^1^TT, concerns emerge regarding SAS for species **B** that would represent the thermalized S_1_. Namely, it is clear that the SAS of **B** is contaminated with spectral features coinciding with ^1^TT. This is most easily seen as ESA or bleach enhancements in **B** (relative to **A**) at all wavelengths where there are strong features in ^1^TT as represented by SAS **C**. While it has been argued that adiabatic electronic states in ultrafast singlet fission systems can take on varying amounts of different electronic character (singlet exciton, charge transfer state, and multiexciton state) during the course of vibronic relaxation,^34,35^ it strikes us as unlikely in this case. First, we see no evidence for systematic ESA spectral shape changes as solvent polarity and polarizability are altered (see Figures S18d, S19c, and S20c), which would be expected to modify the amount of CT character in an adiabatic state. Second, it is difficult to reconcile with 155.1 ps ^1^TT formation if the predecessor state **B** already has substantial ^1^TT character. It appears more likely that the model is incorrect.

An alternative explanation that we favor is that different conformers due to rotation of C–Si bonds exhibit different SF dynamics.^32^ With the notion of multiple conformers existing in photophysical samples, we have sought description of an “averaged” S_1_ lifetime—reflecting different ^1^TT formation time constants—and have employed a stretched exponential function (**A**_stretched_ → **B**) to model the time/wavelength TA matrix for **dimer** in MeTHF. The model is represented by the equation , where τ_relax_ is the lifetime of S_1_ (i.e., **A**), β is a stretching factor representing a distribution of ^1^TT-formation rates among the conformers ranging from 0 (a broad distribution) to 1 (a narrow distribution), and *A*(λ) is a wavelength-dependent ΔOD amplitude. The global fitting is of high quality (Figure S18c), although small residuals remain, likely a result of the nonstatistical (discrete) distribution of possible conformers. The model indicates β= 0.72 ± 0.004 and τ_relax_ = 107.2 ± 0.7 ps, which is reasonably close to 94.3 ps obtained with the single component fit. SAS are shown in Figure S18d and confirm the interpretation that **A** corresponds to S_1_ and **B** to ^1^TT. Further, the fit quality supports the conclusion that only two spectrally distinct species are needed to describe the data. The collection of S_1_ lifetimes for each fitting model can be found in Table S3.

In addition to the solvent MeTHF, TA data were also collected for **dimer** in less polar toluene and more polar benzonitrile. The model **A**_stretched_ → **B** again produces a high-quality representation of data (each with comparable stretching factors of β = 0.7), and we observe a variation in τ_relax_ of 116.6 ± 0.5 ps (toluene), 107.2 ± 0.7 ps (MeTHF), and 76.5 ± 0.3 ps (benzonitrile) (see data, models, and SAS in Figures S18–S20). The result is consistent with participation by a virtual charge transfer state in the electronic coupling mechanism as there are no significant variations in SAS across the solvent. In addition, there is no evidence in the higher polarity medium (e.g., benzonitrile) that a charge transfer state is populated as an intermediate as we and others have observed in other dimer systems.^24,36,37^

### Nanosecond Triplet Pair Evolution

**Figure 4:**
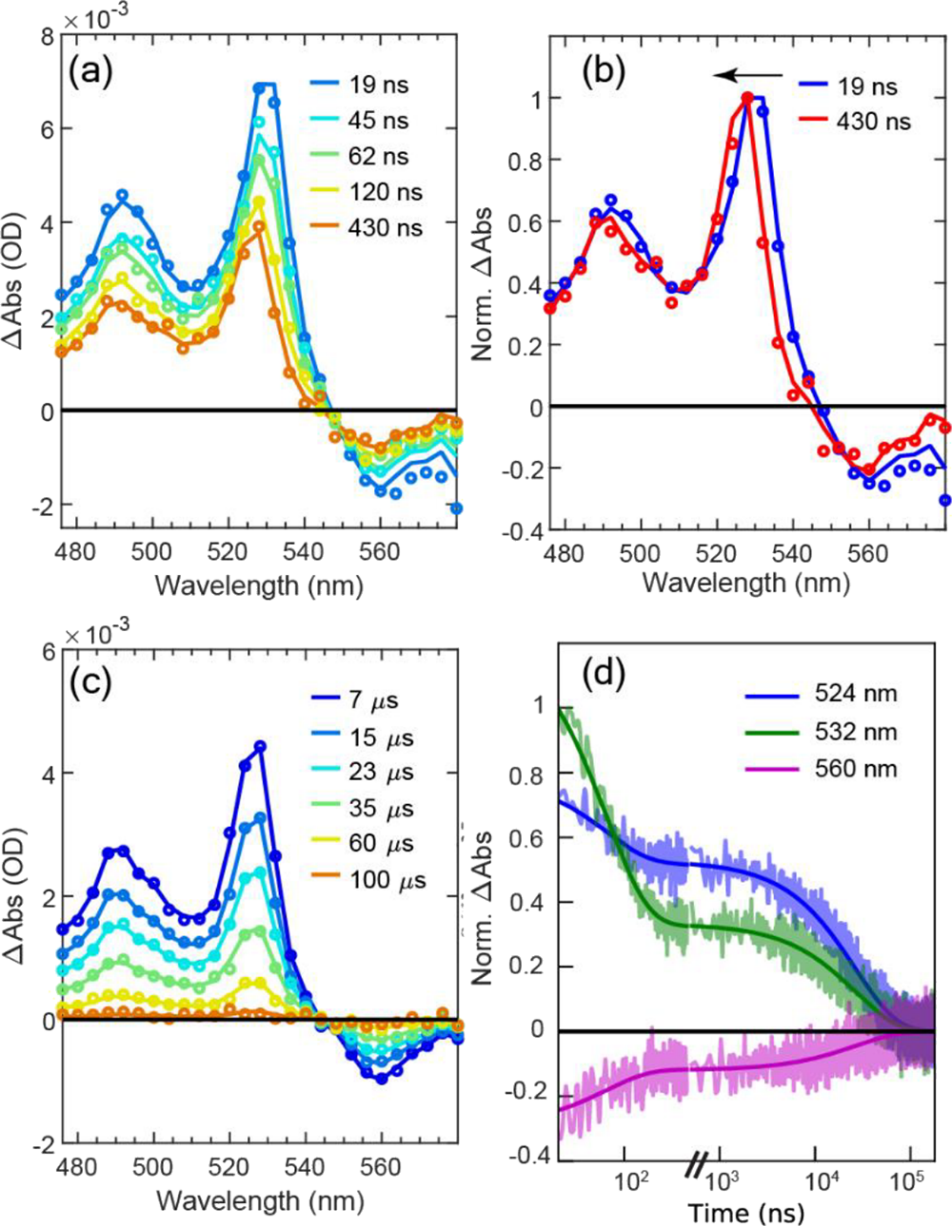
(a) Early time nsTA spectra of **dimer** in MeTHF at room temperature. Opened circles are from raw data. Solid lines are from global fit. (b) Normalized spectra at delay times of 19 and 450 ns from panel (a). (c) Later time nsTA spectra of **dimer** in MeTHF at room temperature. (d) Selected normalized single wavelength decay time traces (transparent lines) from panels (a) and (c) with bold solid lines obtained from global fit.

TA measurements with nanosecond time resolution were used to explore the later time dynamics of the coupled triplet state. An initial data set was collected between 0 and 450 ns with spectral slices shown in Figure 4a and kinetic slices shown in Figure 4d (data before the temporal break). A second data set was then collected for longer times between 450 ns and 100 μs with spectral slices shown in Figure 4c and kinetic slices shown after the temporal break in Figure 4d. In this data set range, the spectral features decay monotonically and a single time constant of 26.6 ± 0.2 μs is sufficient for the model. With this information, it is now possible to model the initial data set using the 26.6 μs time constant as a fixed parameter. This establishes the faster time constant to be 62.5 ± 2.8 ns. In terms of assignments, it is simplest to first consider the slower time constant of 26.6 μs and its corresponding spectrum. These closely match T_1_ generated from a bimolecular sensitization experiment (Figure S28), following photoexcitation of triplet-forming anthracene, and thus, 26.6 μs and its spectrum correspond to the T_1_ of **dimer**.

This then means that the initial 62.5 ns is the time constant required for formation of T_1_ from the multiexciton manifold (^*n*^TT, where *n* = 1, 3, or 5). Interestingly, this occurs concomitantly with both spectral and amplitude changes. For the spectral changes, both the raw (Figure 4b) and fitted (Figures S24 and S25) data indicate an ∼4 nm blue shift in the vibronic peaks as the system evolves. This blue shifting is corroborated by looking at kinetic time traces in Figure 4d: the 532 nm kinetic trace decays more completely in its initial (62.5 ns component) loss compared to a corresponding trace at 524 nm. In terms of amplitude changes, the raw data in Figure 4a suggest a marked decrease in ESA intensity as the system evolves to T_1_. Focusing on a bleach feature to avoid impacts from spectral shifting, the 560 nm kinetic trace (magenta color) in Figure 4d indicates halving of −Δ*A*. This is consistent with the interconversion process: ^*n*^TT → T_1_ + S_0_ during the 62.5 ns process.^27,38^ In other words, isolated triplet (T_1_) is formed but not through decoupling of triplet pairs (^*n*^TT → T_1_ + T_1_). We can use the bleach as a proxy for overall triplet population because weak interchromophore coupling means that ground-state bleach strength associated with T_1_ is half that of TT (c.f., Figure S25, where each species maintains the same ratio of triplet ESA to bleach). This property could in principle allow an estimate of TT formation yield as the bleach evolves on the ps timescale (Figure 3b), but overlapping S_1_ ESA prevents a quantitative analysis.

**Figure 5:**
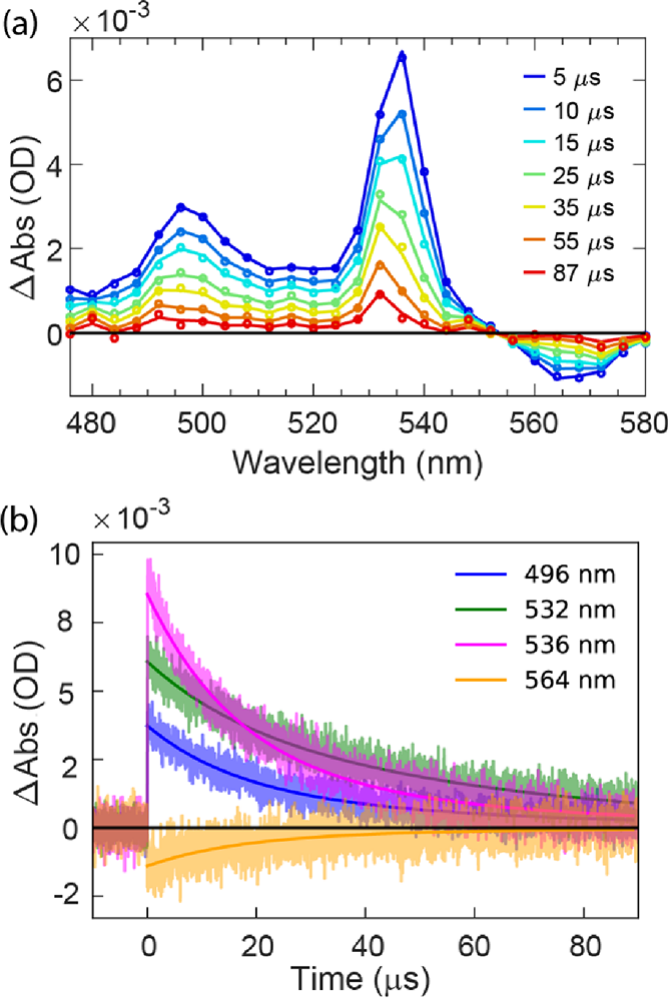
nsTA spectra for **dimer** in MeTHF at 77 K. Opened circles are from raw data. Solid lines are from global fit. (b) Selected single wavelength decay time traces (lightly colored) from panel (a) with bold solid lines from the fit.

Transient absorption data were also collected for **dimer** in optically transparent MeTHF glass at 77 K (Figure 5). Importantly, similar features are observed compared to the room-temperature data described above, namely, spectral blue shifting of the strong vibronic pattern in the visible region and the amplitude halving as the dynamics unfolds. The coupled triplet state (^*n*^TT) evolves to T_1_ with a lifetime of 16.9 ± 0.5 μs. For the longer time constant, 58.1 ± 2.6 μs is observed, which is attributed to the lifetime of T_1_ at 77 K. Compared to the room-temperature data (Figure 4), the decay of ^*n*^TT to T_1_ is ∼300 times slower at 77 K. As a result, at 77 K, the decay of ^*n*^TT and formation of T_1_ are not well separated; the halving of component amplitudes is not apparent in the raw kinetic data (Figure 5b) but is observed in SAS retrieved from global analysis (Figure S26). Again, this can be interpreted as arising as ^*n*^TT converts to a single low energy triplet on the dimer. The 4 nm spectral blue shift in the visible vibronic pattern concurrent with these dynamics is similar at 77 K to what is seen at room temperature (see Figure 5a and Figure S27). The observation confirms that the trEPR data at lower temperature (*vida infra*) will address the same kinds of species being interrogated during room-temperature optical explorations.

For completeness, temperature-dependent early component lifetimes and spectral shifting associated with T_1_ formation were also collected over the range of 298 to 77 K as shown in Figure S23. The fitted ^*n*^TT decay rate coefficients are plotted in an Arrhenius fashion as ln(*k*) versus 1000/*T*, and the trend is complex, in part due to the glass-to-fluid transition of MeTHF at 137 K. In the high-temperature range of 298 to 160 K, approximately linear behavior is observed, indicating an activation energy for the loss of the early time component of 32.3 meV. From 160 to 90K, the decay rate varies significantly, and the trend follows the phase transition of MeTHF, which has a melting point of ∼137 K. In the low temperature range of 90 to 77 K, the slope appears to decrease as thermal energy is lowered, indicating that the dynamics may be entering a regime better described by tunneling, where further cooling cannot extend the early component’s lifetime far beyond 20 μs.

### EPR Spectroscopy

**Figure 6:**
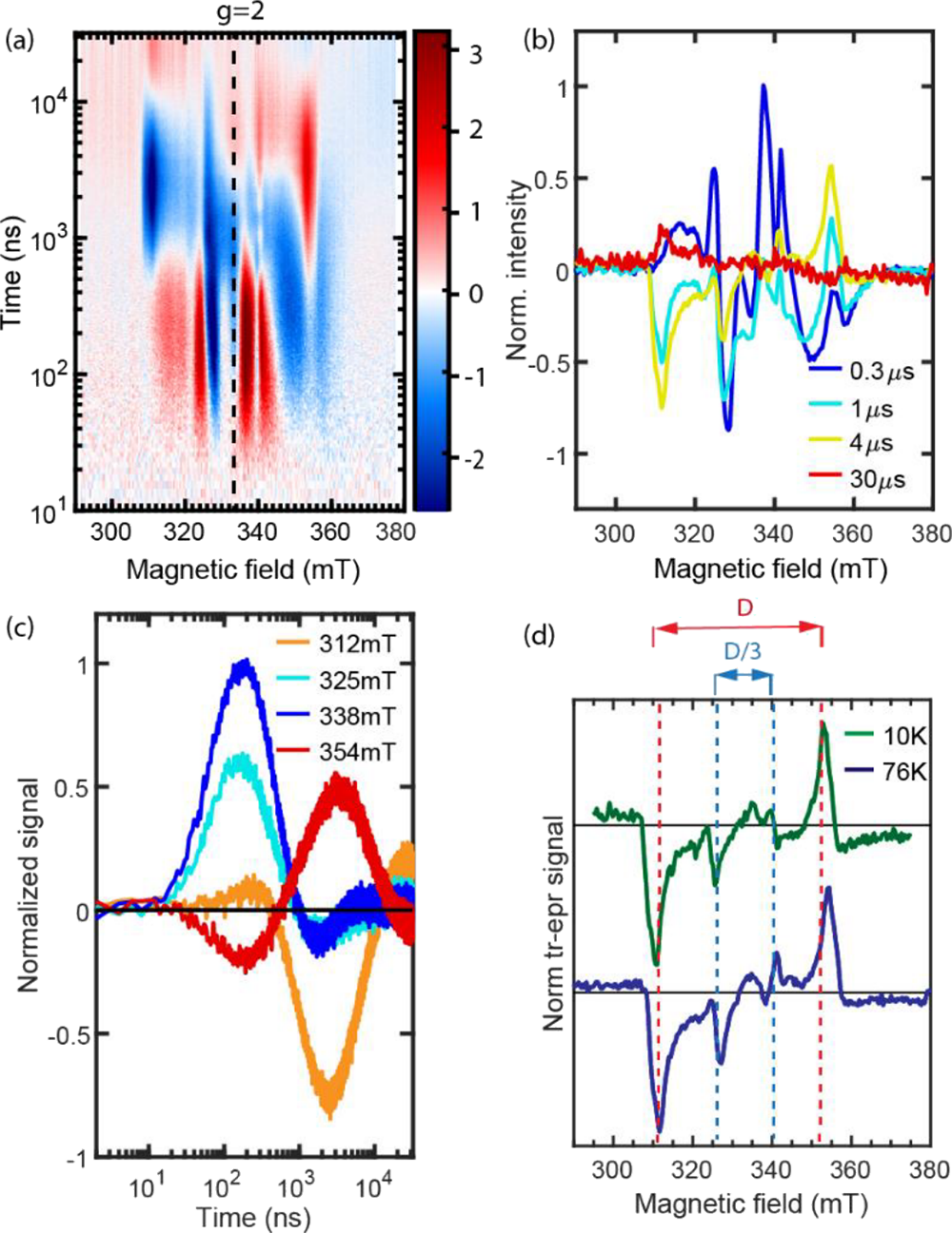
(a) trEPR data for **dimer** in MeTHF at 76 K. (b) EPR spectra for **dimer** in MeTHF at 76 K at the selected delay times. (c) Time traces at 325, 338, 354, and 355 mT. (d) EPR spectrum at 4000 ns measured at 76 K (upper; green) and 10 K (lower; dark blue). The zero-field parameter *D* shown in red approximates splitting between T_±1_ and T_0_. For the quintet transitions, D/3, shown in blue, approximates splitting between ^5^TT_±1_ and ^5^TT_0_.

While the time-resolved TA spectroscopies of previous sections are sensitive to some aspects of the multiexciton dynamics in **dimer**, they do not have sufficient information to assign spin states of the transient species. With this in mind, we conducted both trEPR (transient electron paramagnetic resonance spectroscopy) and pulsed EPR measurements using X-band microwave radiation (9.36 GHz). The trEPR measurements start with an ∼5 ns pulsed optical excitation centered at 608 nm, i.e., into the 0–0 vibronic peak of the S_0_ transition of **dimer**, followed by EPR measurement as a function of time to monitor photoproduct evolution. A pseudocolor plot (magnetic field versus time) showing absorptive (red, Δ*m*_s_ = +1) and emissive (blue, Δ*m*_s_ = −1) magnetic-sublevel transitions for **dimer** in a MeTHF glass at 76 K is shown in Figure 6a.

As seen in Figure 6b, the earliest spectrum shown at 300 ns is complicated, and qualitatively speaking, the strongest transitions are condensed into a narrow portion of the field range (∼325–345 mT). By approximately 1 μs, many of these are lost and a simpler spectrum emerges where the strongest absorptive and emissive transitions are more widely spaced.

The EPR spectral line shape for the coupled triplet state delocalized between the two chromophores depends on the spin-exciton Hamiltonian, which can be written as .^20^ The first term is the isotropic interchromophore exchange interaction between the triplets with A and B representing each chromophore in the dimer. The second term is the anisotropic intrachromophore magnetic dipolar interaction within the triplet. The third term stands for the Zeeman interaction. The second term lifts the degeneracy of nonzero spin manifolds at zero magnetic field, called zero-field splitting, resulting in the separation of the triplet peaks and ^5^TT peaks in EPR spectra. The zero-field splitting parameter, *D*, is a measure of the interaction of unpaired spins of the triplet over the chromophore, and it has been recently reported to be 1273 MHz for T_1_ on **mono** due to ISC in a 4:1 mixture of iodobutane and toluene, as determined using trEPR data.^13^ For **mono** in MeTHF, no EPR active species are observed upon photoexcitation at 608 nm due to the absence of detectable spins. Figure 6d, showing trEPR spectra at a delay time of 4 μs for 76 and 10 K, is annotated with this value of *D* (red dashed vertical lines) according to the equation Δ*B* = *D*/*g*_e_β, where β is the Bohr magneton and using, as an estimate, the gyromagnetic ratio of a free electron (*g*_e_ = 2.0023). The correspondence of *D* with major absorptive and emissive features in the spectra is strong evidence that triplets are formed on the 1–1.5 μs timescale in Figure 6a. Figure 6d has also been annotated with Δ*B* = *D*/3, the expected approximate scale for separation of the most prominent transitions in randomly distributed samples of dimers excited to the ^5^TT state. These trEPR data indicate that the ^5^TT state is the earliest EPR-observable product of SF in **dimer**, consistent with what has been seen in a variety of systems including dimers and films.^13,18,20,21,27,28^ In the SF literature,^14,18,20−22,27,28,39^ an evolution from narrow to wide is interpreted as evidence for conversion from quintet (i.e., ^5^TT) to triplet. Figure 6c shows this development from ^5^TT to triplet: the ^5^TT-related EPR signal at 325 mT (cyan) and 338 mT (blue) rises at ∼300 ns, and while the signal decays, the triplet-related EPR signal at 312 mT (orange) and 354 mT (red) rises accordingly. The rise is apparently nonmonotonic as these outer bands at early times may also have contributions from ^5^TT_±2_. The quintets formed from ^1^TT are expected to show population of these sublevels based upon the principal axes of the constituent chromophore of **dimer** being nonparallel.^18,40^

**Figure 7:**
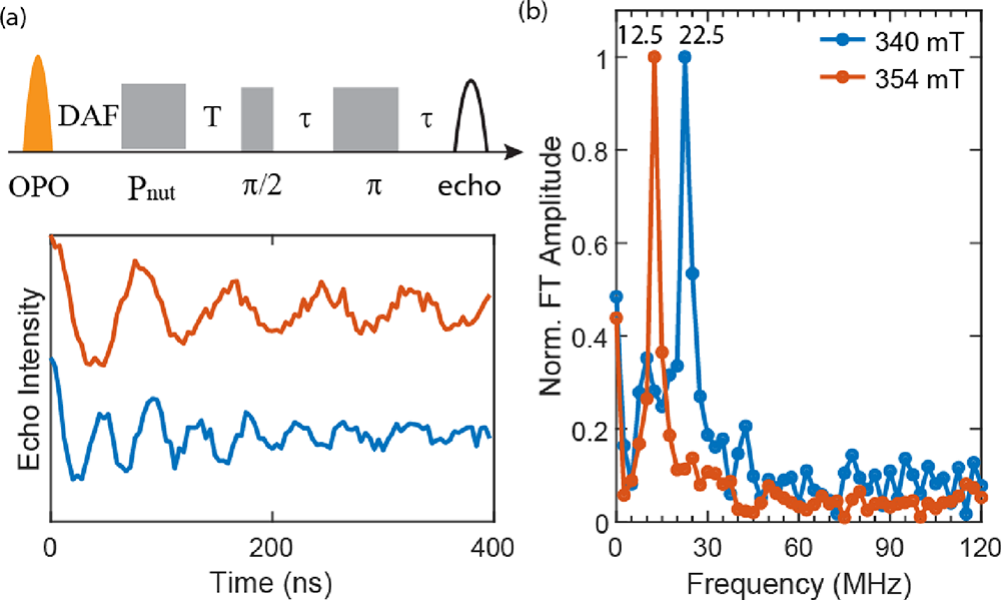
(a) Upper panel: pulse sequence for transient nutation experiment shown in the lower panel, where *T* is 300 ns, τ is 148 ns, and the π pulse length is 32 ns. Lower panel: Rabi oscillations measured at 340 and 354 mT, measured at 10 K in MeTHF. (b) The Fourier transform frequencies of the two Rabi oscillations are 12.5 and 22.5 MHz.

To further support the assignment of long-lived ^5^TT and the triplet features, we conducted pulsed EPR transient nutation experiments initiated at a chosen delay time after optical excitation. In Figure 7a, nutation data are shown for **dimer** at 340 and 354 mT, coincident with the inner and outer sharp features, respectively. For those data, the nutation pulse sequence was initiated 4000 ns after *t*_0_. The nutation frequency depends on both the total spin quantum number, *s*, and specific transition being driven, between *m*_s_ and *m*_s_ ± 1, according to the equation . As can be seen in Figure 7b, the Fourier transform of the time-resolved nutation data indicates ω_340 mT_/ω_354 mT_ = 1.8, which is approximately . Correspondingly, the EPR features at 340 and 354 mT result from *m*_s_ = 0 → 1 transitions within the ^5^TT and triplet manifolds, respectively.

## Discussion

### Formation of ^5^TT

Interpreting the trEPR data, we note that the early time ^5^TT spectra, for example at a delay time of 300 ns in Figure 6b, show an approximately symmetric spectral shape involving opposing absorptive versus emissive sharp features. In the model proposed by Collins et al.,^41^ the exchange energy (*J*) varies in time, which is caused by thermal fluctuations. As *J* ∼ 0, the off-diagonal zero-field splitting term of spin-exciton Hamiltonian becomes important, causing the transition from ^1^TT to ^5^TT. Whether this transition occurs adiabatically depends on the rate of change of *J* from its small isotropic value (*J*_iso_ ∼ *D*) to its maximum value, *J*_max_. This is consequential because a slow *J* fluctuation rate driving ^1^TT to ^5^TT corresponds to net absorptive ^5^TT features, and a diabatic transition (fast *J* fluctuation rate) is associated with symmetric ^5^TT features. We therefore assign our symmetric quintet population to the diabatic regime. Although *J*_max_ of **dimer** is likely to be smaller than that of dimer systems with conjugated bridges that exhibit clear optical signatures of strong coupling, relatively fast motions away from twisted equilibrium geometries and *J*_max_ ≫ *D* should predominantly populate ^5^TT in a symmetric fashion.

In addition to the symmetric line shape, sharp features are seen in other geometrically flexible weakly coupled dimer systems,^25,28^ which could correspond to the contribution of conformers.^25^ The implication that different dimer conformers produce different quintet spectra is consistent with our observations using ultrafast TA spectroscopy (*vide supra*), where a distribution of ^1^TT formation times is found rather than a single well-defined time constant. Distinct ^5^TT spectra associated with unique TES TIPS-TT pairs in the crystal were also inferred.^13^ By contrast, the rigid dimer system TIPS-BP1′^18^ exhibits a much simpler ^5^TT spectrum that is amenable to assignment by rigorous theoretical analysis,^18,40^ taking into account transition probabilities from ^1^TT to the various magnetic sublevel populations of ^5^TT and how these probabilities depend on the orientation of the two chromophore axes relative to one another, as well as the orientation of the dimers within the external magnetic field.

### Coupled Triplet Pair Decay Mechanism

As discussed earlier regarding room-temperature and 77 K nsTA data, 50% ground-state recovery is observed from ^*n*^TT decay (62.5 ns at room temperature and 16.9 μs at 77 K) with the other half following from slow T_1_ decay (26.6 μs at room temperature and 58.1 μs at 77 K). Similar triplet-population halving has been observed in flexible pentacene dimers connected with two and three *p*-phenylene bridges,^27^ twisted pentacene dimers,^15^ and pentacene dimers bridged by two and three tetracene units.^16^ One can expect that ^3^TT → T_1_ + S_0_ internal conversion plays an important role in **dimer**’s population halving dynamics. However, a 16.9 μs lifetime for ^3^TT at 77 K is unlikely for this multiexciton intermediate. Note that in the rigid pentacenic dimer TIPS-BP1′ mentioned above, ^1^TT → S_0_ internal conversion has been observed in ∼150 ns at liquid nitrogen temperatures.^18^ That process is also spin-allowed and nonradiative, but in that case, it releases significantly more energy and likely requires more reorganization energy than for **dimer** undergoing ^3^TT → T_1_ + S_0_. To reconcile the participation of ^3^TT in **dimer** with the apparent sluggishness of the dynamics, we anticipate that ^3^TT formation from other ^*n*^TT multiexciton states is rate-limiting.

With this in mind, and given that the trEPR data confirm that ^5^TT is produced by ^1^TT as seen in other systems as well through spin-mixing,^13,14,18,20,25,27,28,41^ we turn to the question of how ^3^TT connects with ^5^TT. Interconversion/mixing between ^5^TT and ^3^TT is formally forbidden by only considering the magnetic dipolar interaction,^3^ owing to the different polarity of the wave functions. However, system perturbations and dynamics can mitigate this selection rule. For example, Pun et al. have proposed that ^3^TT can rephase from two decorrelated triplets dissociated from ^5^TT.^16^ Also, in the chemically induced dynamic electron polarization (CIDEP) mechanism in singlet fission by Nagashima et al.,^21^ level crossing between the different coupled triplet states is facilitated by triplet migration or molecular motion that changes the distance of the triplets. In **dimer**, which is also conformationally flexible, the process of intersystem crossing from ^5^TT to ^3^TT may be influenced by molecular motion, which is responsive to the phase of the surrounding environment. The relevance of ^3^TT has also been proposed by Chen et al.^39^ in a weakly coupled linear terrylene-3,4:11,12-bis(dicarboximide) dimer (*J* = 20–30 GHz) with evidence of triplet yield enhancement at ^3^TT and ^5^TT level crossing regions.

**Figure 8:**
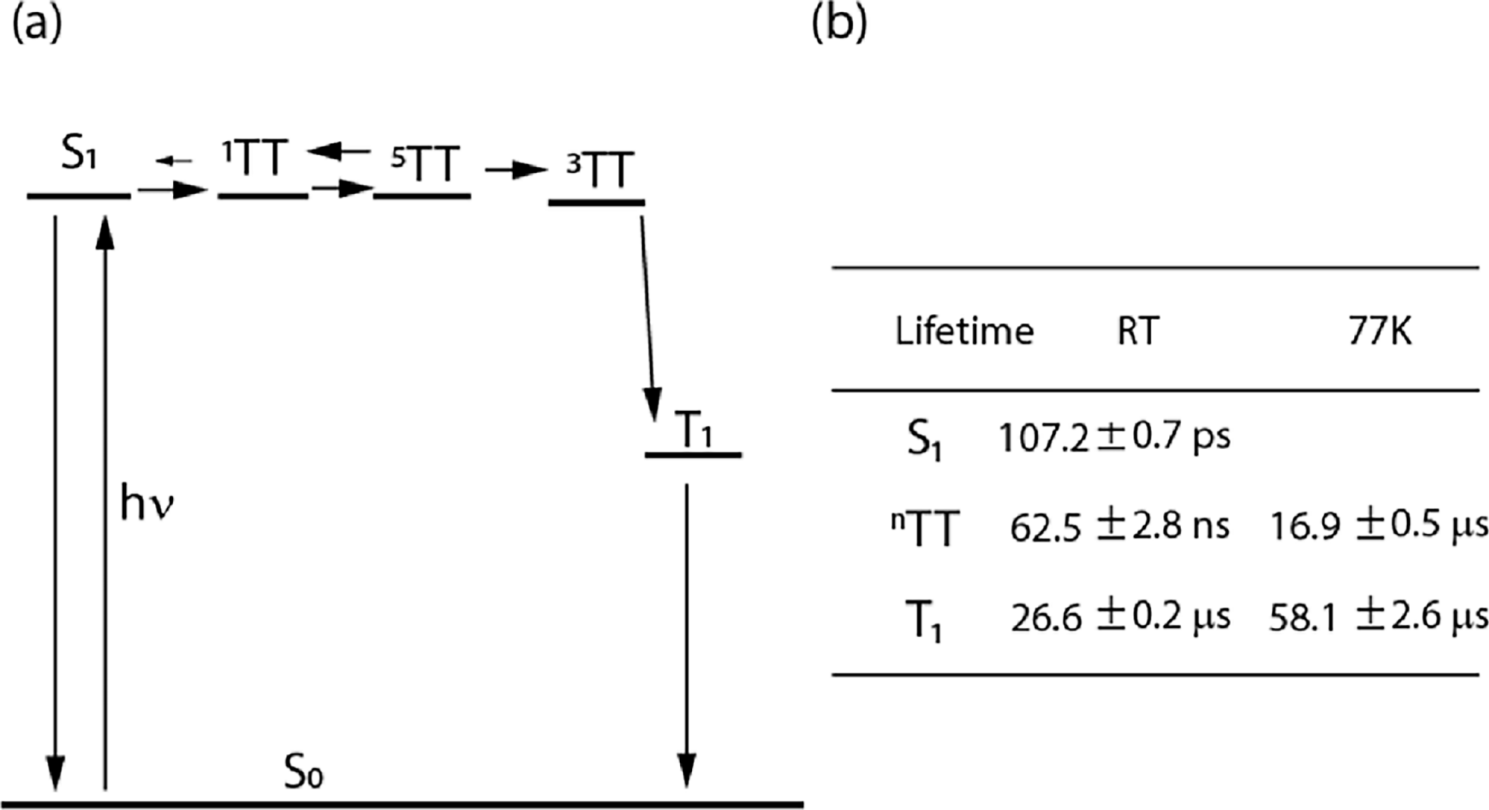
(a) Reaction scheme of dimer upon photoexcitation. (b) Lifetime collection for excited states at room temperature and 77 K.

This hypothesis can be supported by monitoring the decay kinetics of ^*n*^(TT) while gradually reducing the temperature of the system below the melting point of MeTHF. As was seen in Figure 4 at room temperature and Figure 5 at 77 K, the conversion of ^*n*^TT to T_1_ + S_0_ is accompanied by an ∼4 nm blue shift in the peak of the triplet spectrum. Figure S23 confirms that this occurs throughout the cooling profile as seen at 250, 180, 140, and 120 K. While the decay mechanism appears similar during the full temperature range, the coupled triplet decay lifetime (τ_obs_) is sensitive and increases by ∼300 times from 62.5 ns at room temperature to 16.9 μs at 77 K. Visualization of these changes as an Arrhenius plot of ln(*k*_obs_) versus 1/*T* as seen in Figure S23 indicates that the dynamics are coupled to the phase transition of the MeTHF solvent, which has a melting point at 137 K. These data suggest that molecular motions that are more constrained when the liquid solvent transitions to glass at ∼137 K play an important role mediating the interconversion mechanism between ^5^TT and ^3^TT. One possibility is that certain interchromophore motions perturb the spin-exciton Hamiltonian, enabling transient level crossings between ^5^TT and ^3^TT that impact decay dynamics in an average sense. Another possibility is partly based on the perspective of Pun et al.^16^ and considers that *J* fluctuations mediated by the environment and molecular motions manifest in multiexciton dephasing to T_1_ + T_1_. Because the triplets cannot physically separate in a dimer, the two decorrelated T_1_ can subsequently but transiently rephase to form ^3^TT, which would then encounter the internal conversion loss pathway to S_0_ + T_1_. Regardless of the mechanism, ^5^TT converts to ^3^TT with the assistance from molecular motions that are accessible to **dimer** and that are amplified as physical constraints of the glassy solvent are relaxed. The excited state evolution for **dimer** can therefore be proposed in Figure 8a. The lifetime of the excited states at room temperature and 77 K is collected in Figure 8b.

### Triplet in trEPR

**Figure 9:**
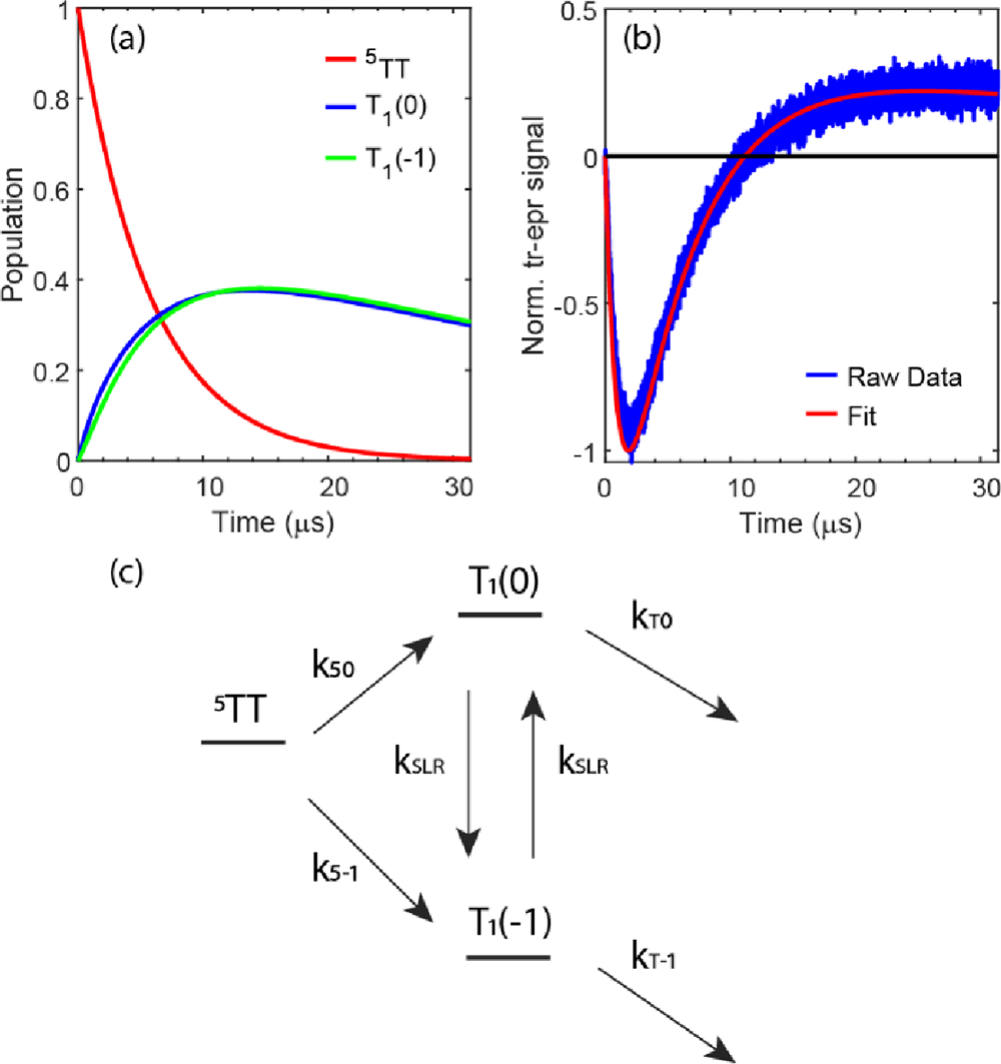
(a) Population analysis from the ODE fit for the trEPR trace at 312 mT. (b) Normalized trEPR trace at 312 mT and the fit from ODE. (c) Kinetic model between ^5^TT, T_1_(0), and T_1_(−1).

A final question involves the nature of the triplets observed at later times in the trEPR spectra of Figure 6, which emerge on a <10 μs timescale but persist for considerably longer, particularly when one takes into account the inflection in the signal sign seen for triplet features occurring at ∼10 μs. As was argued above, it is highly unlikely that ^3^TT would persist on this timescale even at 77 K, given the internal conversion pathway to S_0_ + T_1_. This then suggests that the signals correspond to observation of T_1_. In this section, we apply a simple kinetic model to the triplet trEPR trace at 312 mT to further support this assignment and to explain the observed signal inflection. In our reaction scheme model (shown in Figure 9c^42^), we consider population, depopulation, and spin–lattice relaxation dynamics for three relevant spin states, ^5^TT, T_1_(0), and T_1_(1), where the number in the parentheses represents the spin quantum number, *m*_s_. *k*_50_ and *k*_5–1_ are the population rate coefficients from ^5^TT to T_1_(0) and to T_1_(−1), respectively, while *k*_T0_ and *k*_T–1_ are depopulation rate coefficients from T_1_(0) and T_1_(−1), respectively. Finally, *k*_SLR_ is the spin–lattice relaxation rate coefficient between T_1_ spin states. The normalized trEPR trace at 312 mT along with the fit from ordinary differential equations (ODEs) based on our kinetic scheme is shown in Figure 9b. The population analysis from the fit can be seen in Figure 9a. The fitting result suggests τ_50_ = 8.5 μs, τ_5–1_ = 17.5 μs, τ_T0_ = 29 μs, τ_T–1_ = 187 μs, and τ_SLR_ = 1.94 μs. These fitting results suggest that the population of T_1_(0, blue) and T_1_(−1, green) crosses at 11.3 μs as a combined effect from faster population but also faster depopulation of T_1_(0) compared to T_1_(−1), which is also seen in other pentacene systems.^27,43^ This relative population shift then manifests in the sign change at 11.3 μs for the normalized trEPR at 312 mT in Figure 9b. Notably, the depopulation time constants for ^5^TT from the ODE fit (τ_50_ = 8.5 μs and τ_5–1_ = 17.5 μs) closely align with the initial decay component from the cryo-nsTA experiment (16.9 μs; Figure 5). The depopulation lifetimes of T_1_(0) and T_1_(−1) (τ_T0_ = 29 μs and τ_T–1_ = 187 μs) agree less closely with the T_1_ lifetime, 58.1 μs, extracted from cryo-nsTA, although they share the same order of magnitude. Here, however, there is limited data leading to significant uncertainty in determination of the longer τ_T–1_ time constant.

## Conclusions

In summary, we have identified **dimer**, Et_2_Si(TIPSTT)_2_, to be weakly coupled electronically, and its SF mechanism has also been revealed. This system shows fast evolution from S_1_ to ^1^TT with high yield, and ^1^TT proceeds toward spin-mixing with other TT states owing to the weak coupling between the chromophores. In addition, the halving of the coupled triplet pair population to T_1_ strongly supports a route through ^3^TT. Based on the results from the kinetic fit of the trEPR trace and cryo-nsTA, we demonstrate that the kinetics shown in trEPR is related to the evolution from ^5^TT to T_1_, implying ^3^TT as an unobserved ephemeral intermediate. Even though the conversion between ^5^TT and ^3^TT is forbidden via dipolar interaction, molecular motion can facilitate the process. The diabatic nature of ^5^TT spin sublevel population indirectly suggests significant interchromophore motions, and the solvent phase-dependent decay rate from TT to T_1_ further implicates molecular motion as an essential key to allow the originally forbidden process. Ultimately, the full excited state evolution for **dimer** is S_0_S_0_ → S_0_S_1_ ⇌ ^1^TT ⇌ ^5^TT → ^3^TT → T_1_ + S_0_ → S_0_ + S_0_.

The mechanism highlights a challenge with this dimer molecule, as it forms only one triplet exciton from a single photon excitation, subverting the prime advantage of singlet fission for applications such as photovoltaics (although even with less than 100% yield, spin-polarized triplets could still be of interest for other applications). To minimize the pathway leading to single triplet formation and maximize the yield of triplets for SF-based solar cell application, it is necessary to inhibit the formation of ^3^TT. The most direct strategy is through the addition of chromophores beyond a dimer to introduce a fast triplet hopping pathway in the system^44^ that outcompetes evolution to ^3^TT. For improving free triplet yields in the dimer itself, reducing the likelihood of symmetry-breaking motions that facilitate ^3^TT formation could be achieved by rigidifying or sterically encumbering the structure, such as by modifying the bridge or encapsulating the dimers in a film or matrix.

## Data Availability

The data underlying this study are available in the published article and its Supporting Information.

## Supporting Information Available

NMR data, X-ray diffraction experiment details, and additional spectroscopic and EPR experiments (PDF)
