# Supplementary material for: Revealing the Singlet Fission Mechanism for a Silane-Bridged Thienotetracene Dimer
Source: J Phys Chem A. 2024 May 8;128(20):3982–92. doi: 10.1021/acs.jpca.4c01463 (PMC11129308; doi:10.1021/acs.jpca.4c01463)
Supplement: Supplementary file 1 — jp4c01463_si_001.pdf [file jp4c01463_si_001.pdf]

Supporting information for

Revealing the singlet fission mechanism for a silane-bridged thienotetracene dimer

Liang-Chun Lin[1], Ryan D. Dill[1], Karl J. Thorley[2], Sean R. Parkin[2], John E. Anthony[2], Justin C. Johnson*[3,4], Niels H. Damrauer*[1,4]

1. Department of Chemistry, University of Colorado Boulder, Boulder, CO 80309, USA.
2. Department of Chemistry & Center for Applied Energy Research, University of Kentucky, Lexington, Kentucky 40506-0055, USA.
3. National Renewable Energy Laboratory, 15013 Denver West Parkway, Golden, Colorado 80401, USA.
4. Renewable and Sustainable Energy Institute (RASEI), University of Colorado Boulder; Boulder, CO, 80309, USA.

Contents

1. **Synthesis and Characterization** 3
2. **Experimental Methods** 4
   2-1. Femtosecond transient absorption (fsTA) spectroscopy. 4
   3-2. Nanosecond transient absorption (nsTA) spectroscopy. 4
   3-3. Time-Correlated single photon counting (TCSPC) 5
   3-4. Electron paramagnetic resonance (EPR) 5
3. **Steady-State Absorption and Emission** 6
   3-1. Steady-state UVVis absorption spectra of mono and dimer in chloroform 6
   3-2. Steady-state UVVis absorption and emission spectra of dimer in toluene and MeTHF 7
   3-3. Steady-state UVVis absorption and emission spectra of mono and dimer in MeTHF 8
4. **Optimized geometries of $Et_2Si(SiH_3TT)_2$ for three conformers** 11
5. **TCSPC measurement - dimer** 13
6. **Ultrafast TA fitting - dimer** 15
   6-1. A→B→0 model 15
   6-2. A→B→C→0 model 16
   6-3. A (stretched)→B→0 model 17
7. **Solvent dependent singlet fission of dimer via fs-TA** 19
   7-1. dimer in toluene 19
   7-2. dimer in benzonitrile 20
8. **mono fsTA & nsTA & TCSPC measurements** 21
   8-1. mono fsTA 21
   8-2. mono nsTA and TCSPC 22
9. **dimer temperature dependent nsTA** 23
10. **Species associated spectra (SAS)** 24
   10-1. SAS for nsTA at room temperature 24
   10-2. SAS for nsTA at 77 K 25
11. **Triplet sensitization of mono** 27
12. **trEPR and pulsed EPR measurement on dimer at 10K** 28
13. **Emission quantum yield measurement** 28

Reference 30

1. Synthesis and Characterization

Anhydrous tetrahydrofuran and lithium diisopropylamide were purchased from Sigma Aldrich. Dichlorodiethylsilane was purchased from TCI. Hexanes, dichloromethane and tetrahydrofuran were purchased from VWR. Silica for chromatography (40-63 μm, 60 Å) was purchased from Silicycle. Size exclusion chromatography was performed using an SX-1 stationary phase from Bio-Rad, with tetrahydrofuran as the eluent under ambient pressure. All purchased chemicals were used without further purification. TIPSTT [1]and TES-TIPSTT[2]were prepared according to literature procedures.

Proton and carbon NMR spectra were collected using a 400 MHz Bruker spectrometer. Chemical shifts of each spectrum are reported in ppm and referenced to deuterated chloroform (Sigma Aldrich) solvent. HRMS was measured using a ThermoFisher Q-Exactive spectrometer by ESI in positive mode using a 10 μg/mL solution in 1:1 acetonitrile/water.

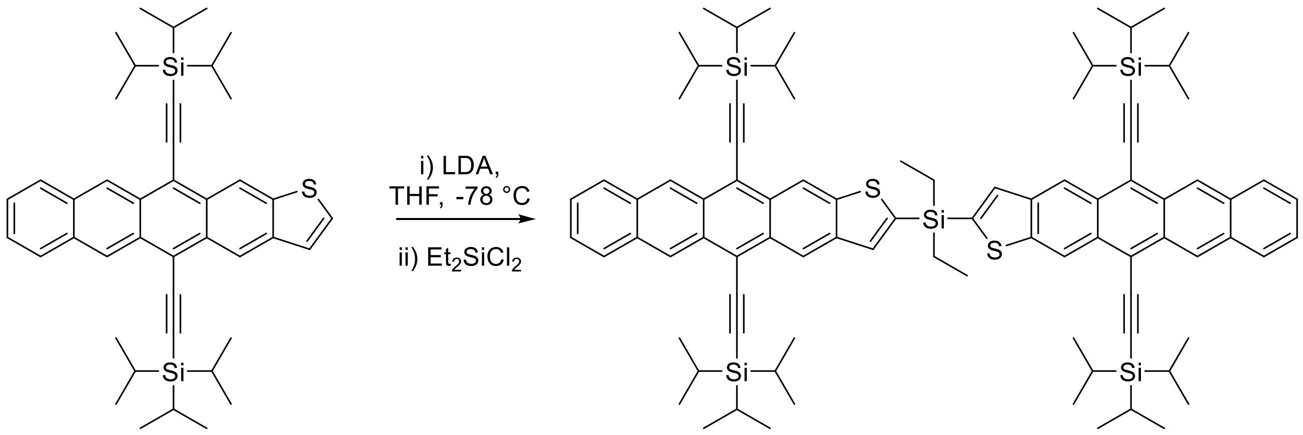

TIPS-TT (0.400 g, 0.62 mmol) was dissolved in anhydrous THF (100 mL) and cooled to -78 °C. Lithium diisopropylamide (1M in THF, 0.93 mL, 0.93 mmol) was added, and the mixture stirred for 15 minutes. Dichlorodiethylsilane (0.44 mL, 3.1 mmol) was dissolved in 20.0 mL of anhydrous THF. 2.05 mL of this solution (0.31 mmol) was added dropwise, and the reaction stirred for 16 hours at room temperature. The reaction was quenched with H_2O (25 mL) and then 10 % HCl solution (25 mL). The product was extracted with CH_2Cl_2 (50 mL) and washed with H_2O (50 mL). The solvent was removed and the crude product passed through a silica plug (hexanes). The mixture was then further purified by size exclusion chromatography (SX-1, THF) and silica column (hexanes). Recrystallisation from 2-butanone gave $Et_2Si(TIPSTT)_2$ as dark purple crystalline blocks (0.11 g, 26 %).

^{1}H NMR (400 MHz, $CDCl_3$) δ 9.33 (4H, d, J = 5.0 Hz), 9.19 (4H, d, J = 8.7 Hz), 8.00 (4H, dd, J = 6.2, 2.9 Hz), 7.74 (2H, s), 7.44 (4H, dd, J = 6.8, 3.4 Hz), 1.27-1.40 (99H, m)

^{13}C NMR (100 MHz, $CDCl_3$) δ 142.6, 141.5, 141.3, 133.3, 132.2, 132.1, 130.5, 130.4, 130.2, 128.6, 128.6, 126.3, 126.2, 126.0, 125.9, 121.7, 119.6, 119.0, 117.4, 106.8, 106.4, 104.4, 104.4, 19.0, 19.0, 11.7, 7.5, 5.3

HRMS (ESI, +ve) m/z $C_{88}H_{112}S_2Si_5$ requires 1372.705184 observed 1372.6955

Copies of Spectra

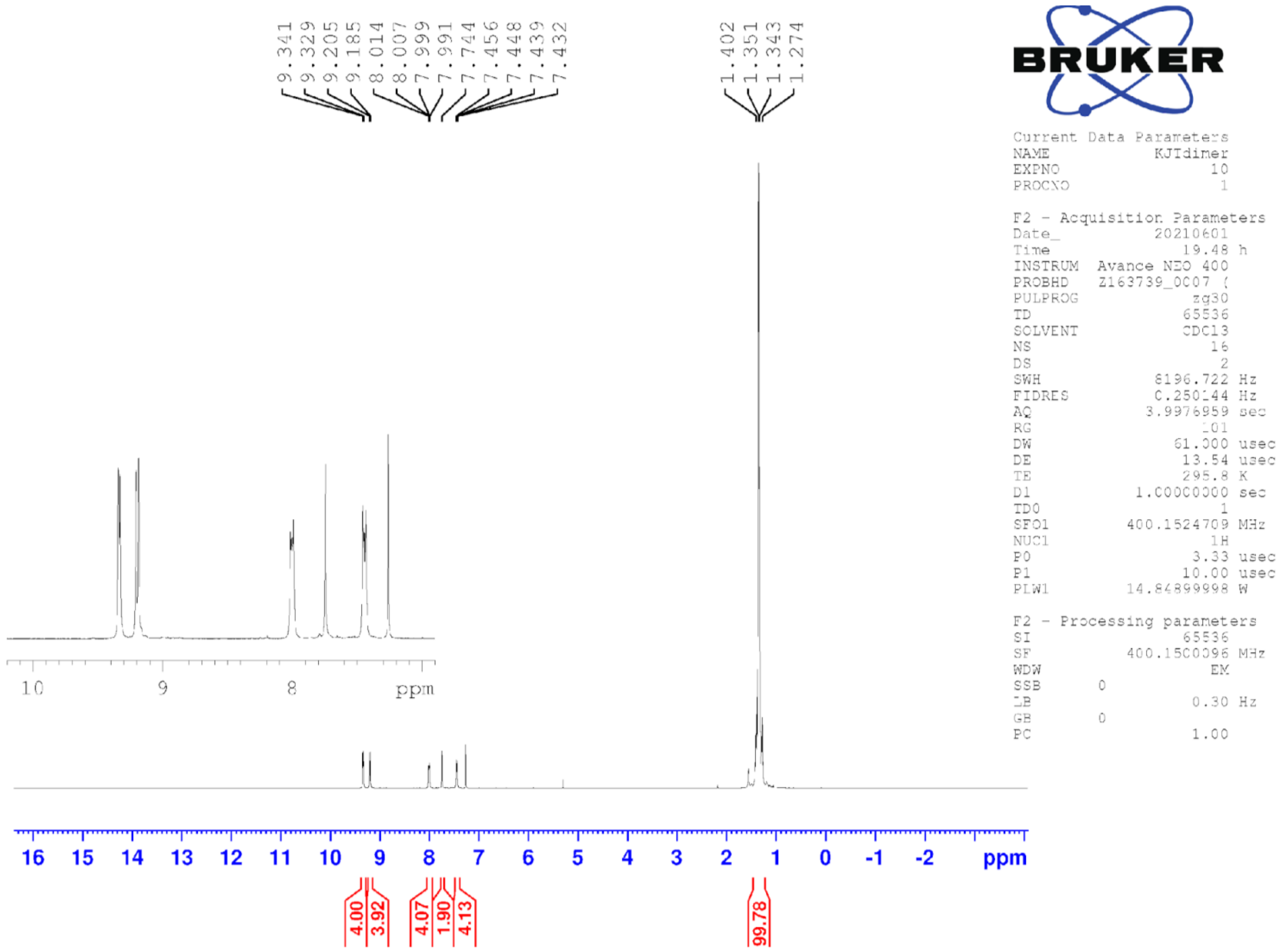

Figure S1. 400 MHz ^{1}H NMR spectrum of $Et_2Si(TIPSTT)_2$ dimer measured at 298 K in $CDCl_3$

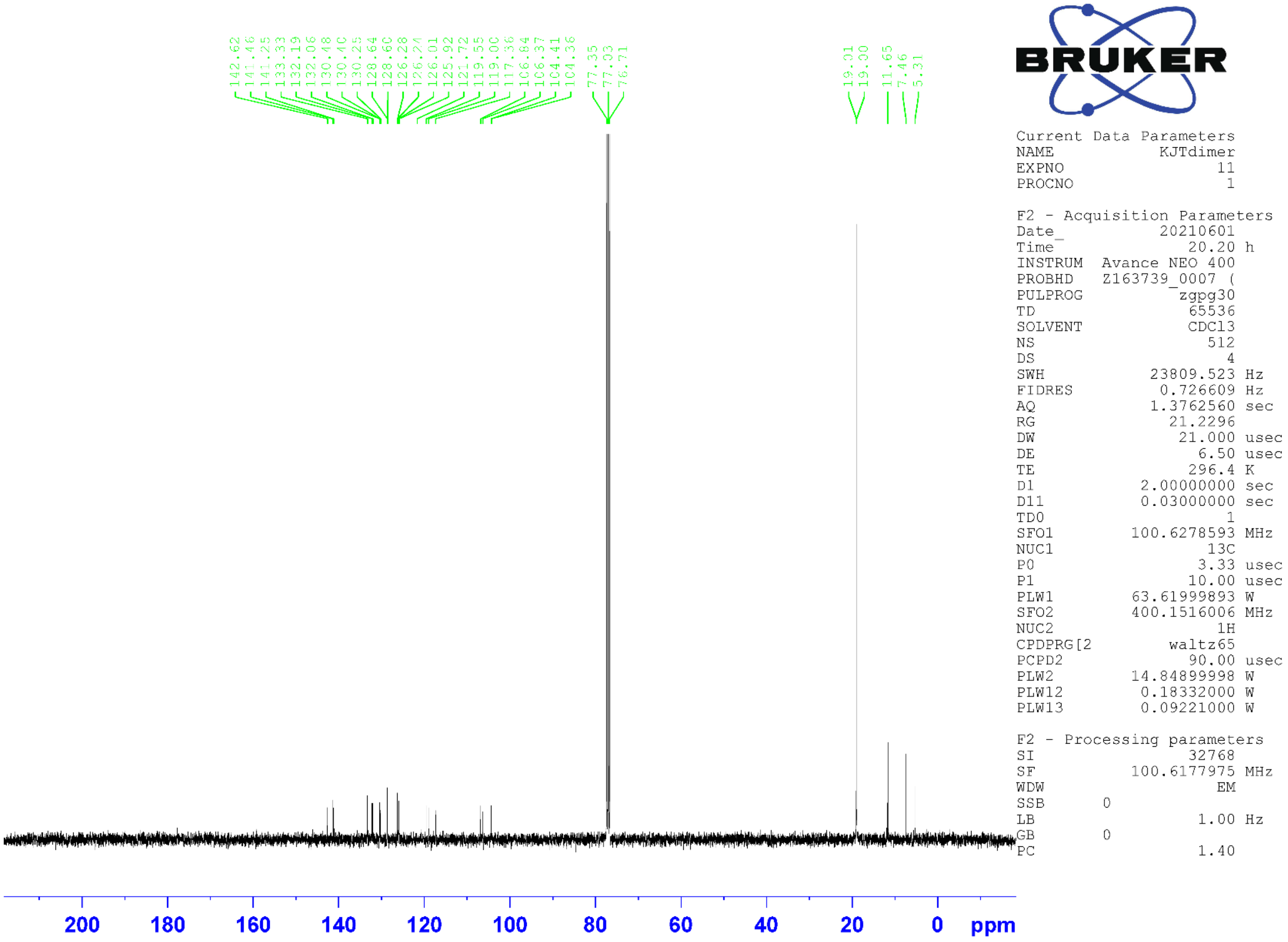

Figure S2. 100 MHz ^{13}C NMR spectrum of $Et_2Si(TIPSTT)_2$ measured at 298 K in $CDCl_3$

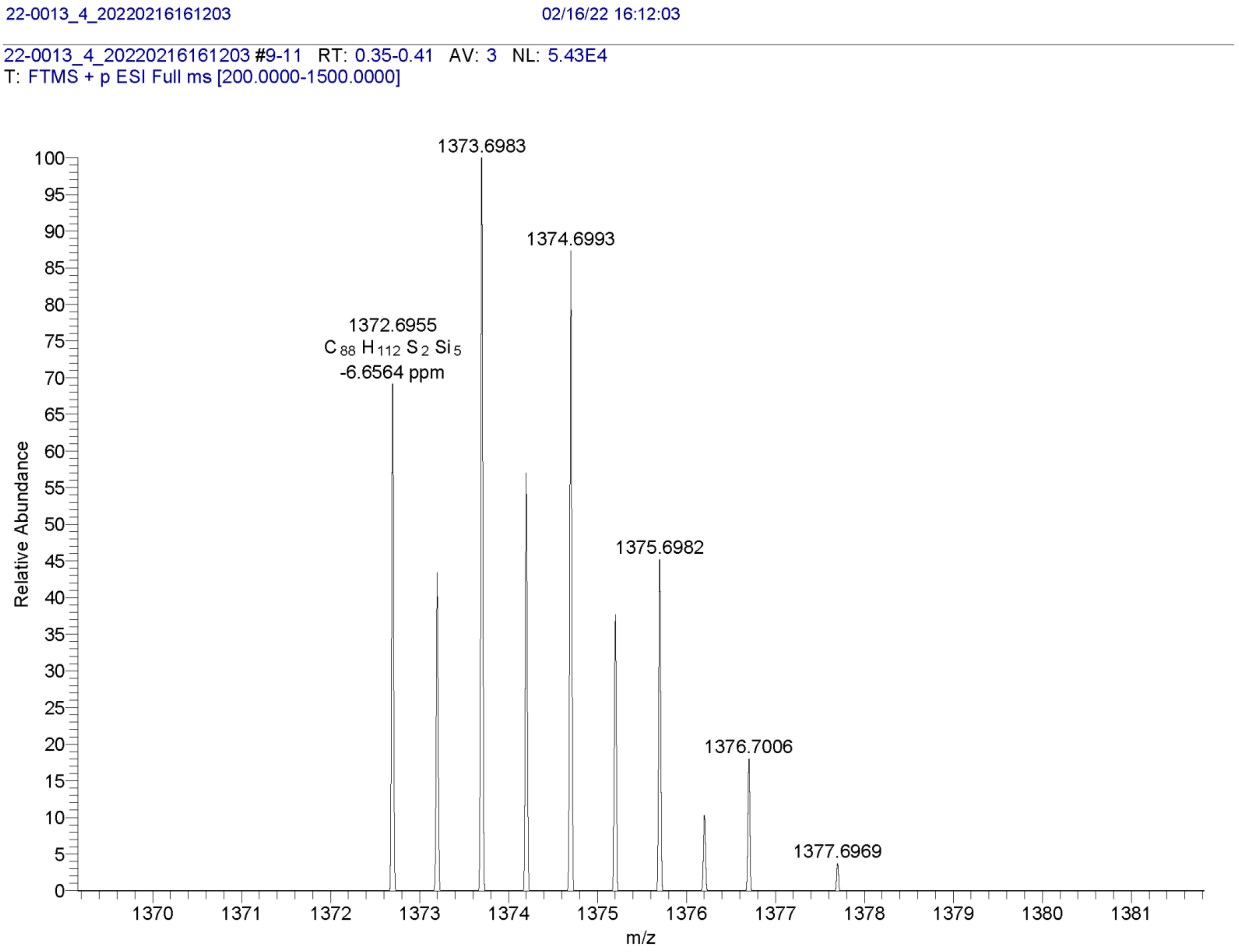

Figure S3. High resolution ESI mass spectrum of $Et_2Si(TIPSTT)_2$

X-ray diffraction data

X-ray diffraction data was collected at 90.0(2) K on a Bruker D8 Venture dual-source diffractometer with graded-multilayer focused MoK(alpha) X-rays. Raw data was integrated, scaled, merged, and corrected for Lorentz-polarization effects using the APEX3 package (Bruker-AXS,2018). Corrections for absorption were applied using SADABS[3]. The structures were solved by dual-space methods (SHELXT)[3] and refined against F^2 by weighted full-matrix least-squares (SHELXL-2018[4]). Hydrogen atoms were found in difference maps but subsequently placed at calculated positions and refined using riding models. Non-hydrogen atoms were refined with anisotropic displacement parameters. The final structure model was checked using established methods[5,6]. Atomic scattering factors were taken from the International Tables for Crystallography[7].

Table 1. Crystal data and structure refinement for m21103.

Identification code	m21103
Empirical formula	C88 H112 S2 Si5
Formula weight	1374.34
Temperature	90.0(2) K
Wavelength	0.71073 A
Crystal system, space group	Triclinic, P-1
Unit cell dimensions	a = 9.2637(9) A alpha = 84.197(3) deg.
	b = 12.4736(13) A beta = 83.254(4) deg.
	c = 37.449(4) A gamma = 74.162(4) deg.
Volume	4123.4(7) A^3
Z, Calculated density	2, 1.107 Mg/m^3
Absorption coefficient	0.179 mm^-1
F(000)	1484
Crystal size	0.400 x 0.300 x 0.010 mm
Theta range for data collection	1.957 to 25.249 deg.
Limiting indices	-10<=h<=11, -14<=k<=14, -44<=l<=44
Reflections collected / unique	74209 / 14043 [R(int) = 0.0492]
Completeness to theta = 25.242	94.4 %
Absorption correction	Semi-empirical from equivalents
Max. and min. transmission	0.971 and 0.846
Refinement method	Full-matrix least-squares on F^2
Data / restraints / parameters	14043 / 984 / 922
Goodness-of-fit on F^2	1.159
Final R indices [I>2sigma(I)]	R1 = 0.0883, wR2 = 0.1699
R indices (all data)	R1 = 0.1188, wR2 = 0.1796
Extinction coefficient	n/a
Largest diff. peak and hole	0.327 and -0.341 e.A^-3

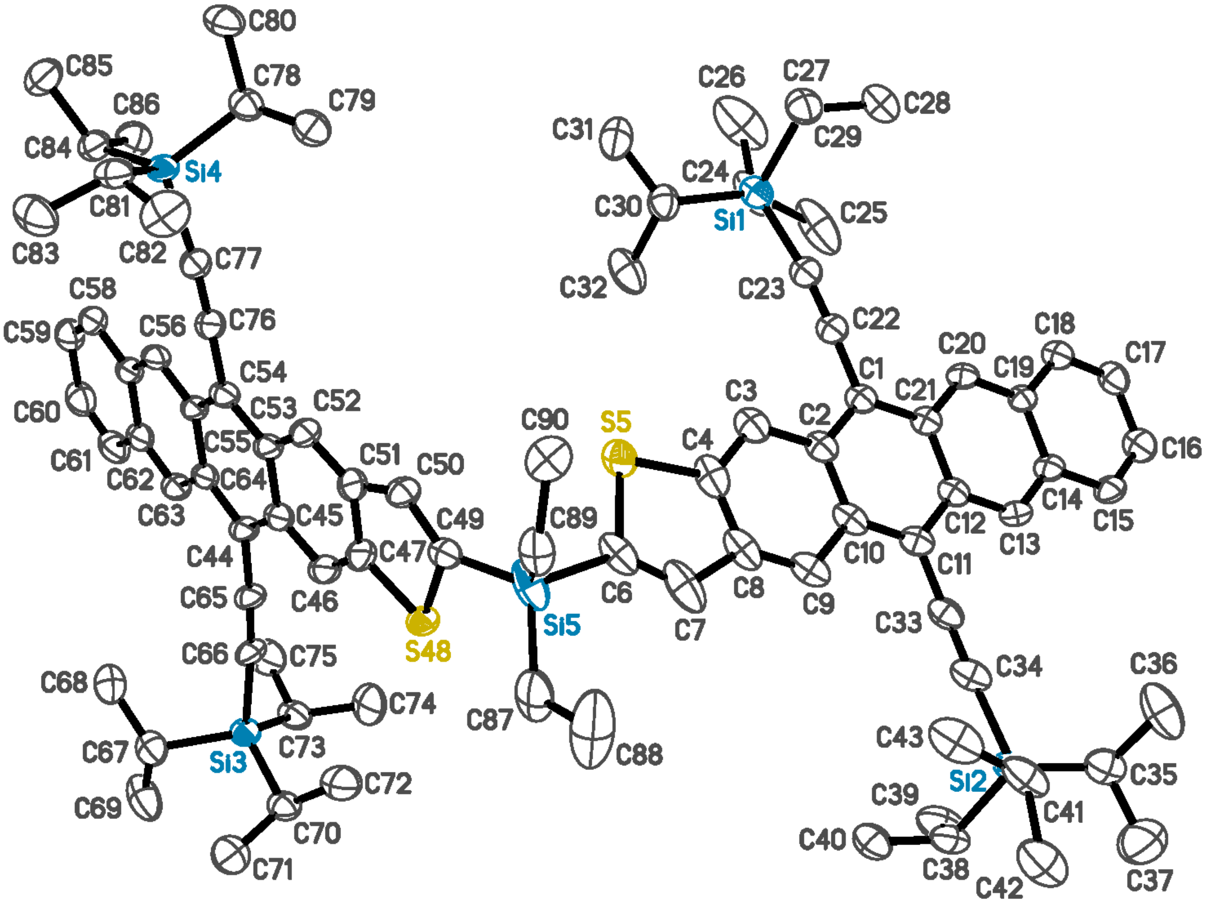

Figure S4. Ellipsoid plot (50%) for $Et_2Si(TIPSTT)_2$

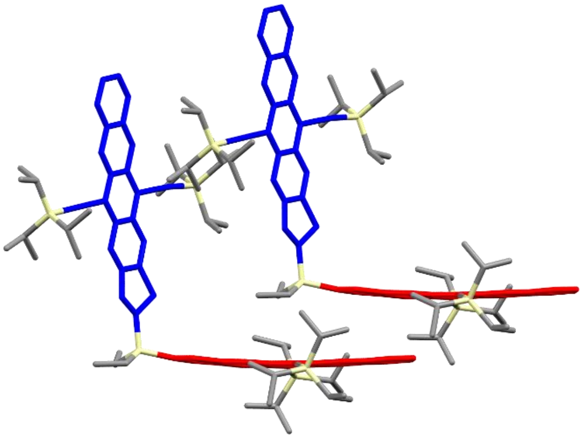

Figure S1. Pair of nearest neighbors from the crystal structure of $Et_2Si(TIPSTT)_2$

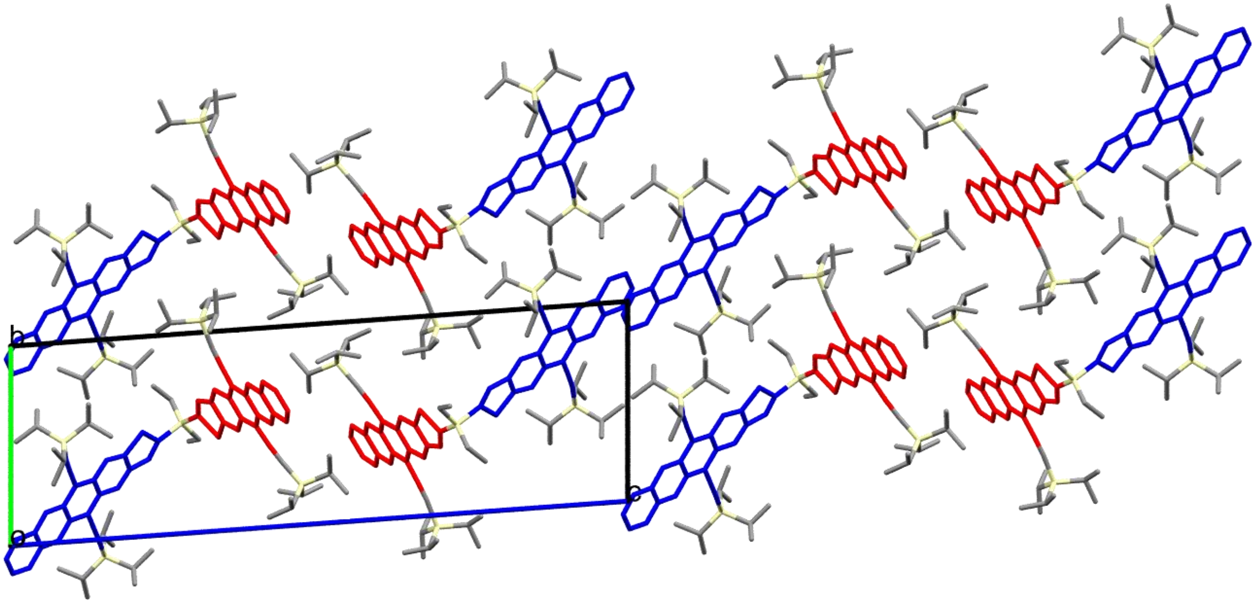

Figure S2. View of the crystal structure of $Et_2Si(TIPSTT)_2$ along the crystallographic a axis

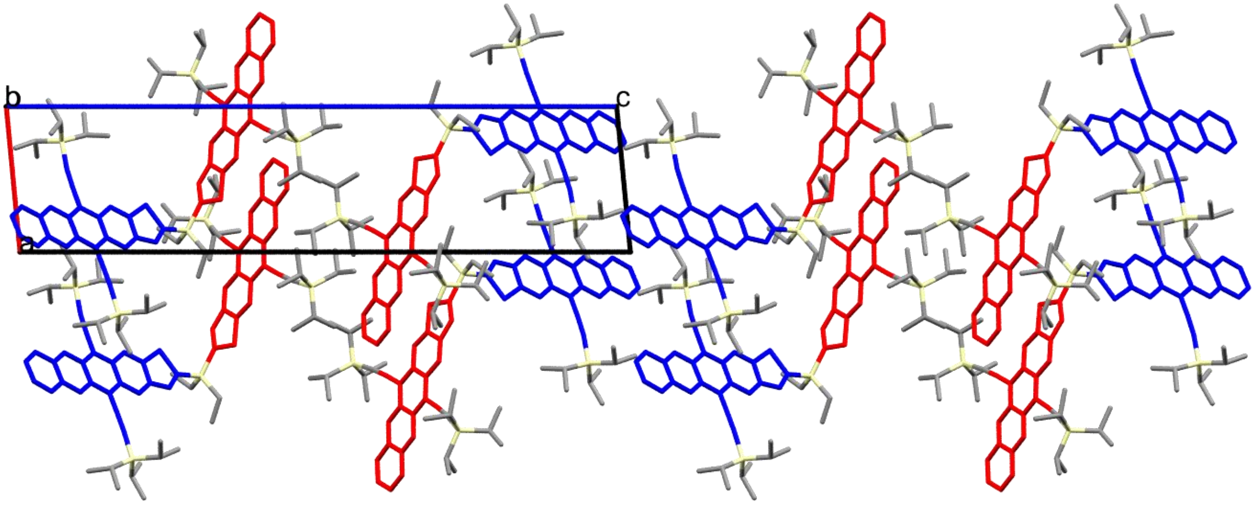

Figure S3. View of the crystal structure of $Et_2Si(TIPSTT)_2$ along the crystallographic b axis

2. Experimental Methods

2-1. Femtosecond transient absorption (fsTA) spectroscopy.

The femtosecond transient absorption spectra were measured with a home-built pump probe setup. 2nd harmonic of CW Nd:YVO4 laser (Coherent Verdi) pumped Ti:Sapphire oscillator (KM Labs) to generate pulses centered at ~ 800 nm with ~50 fs pulse duration at 94 MHz repetition rate. The multi-pass amplifier (Quantronix Ordin) amplified the 800 nm pulse to a pulse energy ~1mJ/pulse. For the pump beam, the amplified 800 nm pulses were sent into a home-built non-collinear optical parametric amplifier (NOPA), which output pulses centered at 604 nm with FWHM ~40 nm. The prism compressor downstream compressed the NOPA output pulse to a pulse duration 35-50 fs. The pump beam was chopped at 350 Hz and focused on the sample with magic angle (54.7^{o}) polarization relative to the probe beam. The diameter of pump beam at the sample were measured to be ~ 200 um FWHM and the pulse energy was measured to be ~100-200 uJ/cm^2.

For probe beam, the continuum white light pulses were generated by elliptically translated CaF_2 promptly after 10% amplified 800 nm pulse being focused on. The transmitted probe beam was focused into Chromex 25-IS spectrograph before 350 Hz detection with Andor Newton camera. The samples were prepared in 2 mm quartz cuvettes equipped with Kontes Hi-VAC (R) vacuum valves. We used high purity 2-MethylTHF (BioRenewable, anhydrous, ≥99%, Inhibitor-free) to dissolve the Et2SiTIPSTT solid into 23 μM solution in glovebox.

3-2. Nanosecond transient absorption (nsTA) spectroscopy.

The nanosecond transient absorption spectra were measured with a home-built pump-probe setup. The source laser pulses were generated from combination of Nd:YAG pump (Continuum Surelite II) and optical parametric oscillator (OPO, Continuum Surelite II). 355 nm pulses were derived from Nd:YAG pump at repetition rate of 10 Hz and were sent to the OPO at the downstream to generate a tunable output pulse with a range from 400 nm to 700 nm. The pulse duration is measured to be ~5 ns. We attenuated the pulse centered at 604 nm to a power read from 200-250 uW to the sample. Xeon lamp generated a broadband probe that was focused on the sample via a plano convex lens (f=75 mm) at 90-degree incident angle from the pump beam. The transmitted white light was focused to monochromator with a resolution of +/-1.7 nm. We obtained signal from PMT (Hamamatsu, R928-07), biased negatively at -1000V via oscilloscope (Lecroym

LC584AL). Transient decay time traces were fitted with single or double exponential decay function.

The samples were prepared in 1cm quartz cuvettes equipped with Kontes Hi-VAC (R) vacuum valves. We used high purity 2-MethylTHF (BioRenewable, anhydrous, ≥99%, Inhibitor-free) to dissolve the Et2SiTIPSTT solid into 4.6 µM solution in glovebox.

3-3. Time-Correlated single photon counting (TCSPC)

The experiments were conducted with Delta Flex Modulator fluorescence lifetime system from Horiba scientific. The source laser pulses were derived from Horiba NanoLED-405L (402 nm CWL, 200 ps pulse duration). The temperatures were regulated by VWR 1145 Refrigerated Constant Temperature Circulator via a jacket cuvette holder, where the temperature-controlled water circulated. The Emission traces were fitted with singlet exponential decay function.

3-4. Electron paramagnetic resonance (EPR)

EPR experiments were performed with a commercially available spectrometer (Bruker Elexsys E580) quipped with dielectric resonator, Bruker ER4118 X-MS3. Singlet fission is driven by the pulsed laser excitation (Wavelength centered at 605 nm, 10 Hz repetition rate, pulse energy ~ 3.5 mJ, ~5 ns FWHM) was provided by a fiber-coupled optical parametric oscillator (Opotek Radiant SE 355 LD). The beam diameter at the sample is approximately 8.5 mm. The microwave frequency is centered at 9.36 GHz. The attenuation of the microwave is set to be 17 dB, which is ~3 mW for trEPR measurement. The detailed sample preparation procedure can be found in Dill 2023[9]. For pulsed EPR experiment, the transient nutation traces are obtained with a pulse sequence of $P_{nut} - T - \frac{\pi}{2} - \tau - \pi$, where P_{nut} is variable, T is 300 ns, τ is 148 ns, π pulse length is 32 ns with microwave power set to be 20 dB, which is optimized to the ideal tuning angle for the quintet spin echo.

3. Steady-State Absorption and Emission

3-1. Steady-state UVVis absorption spectra of mono and dimer in chloroform

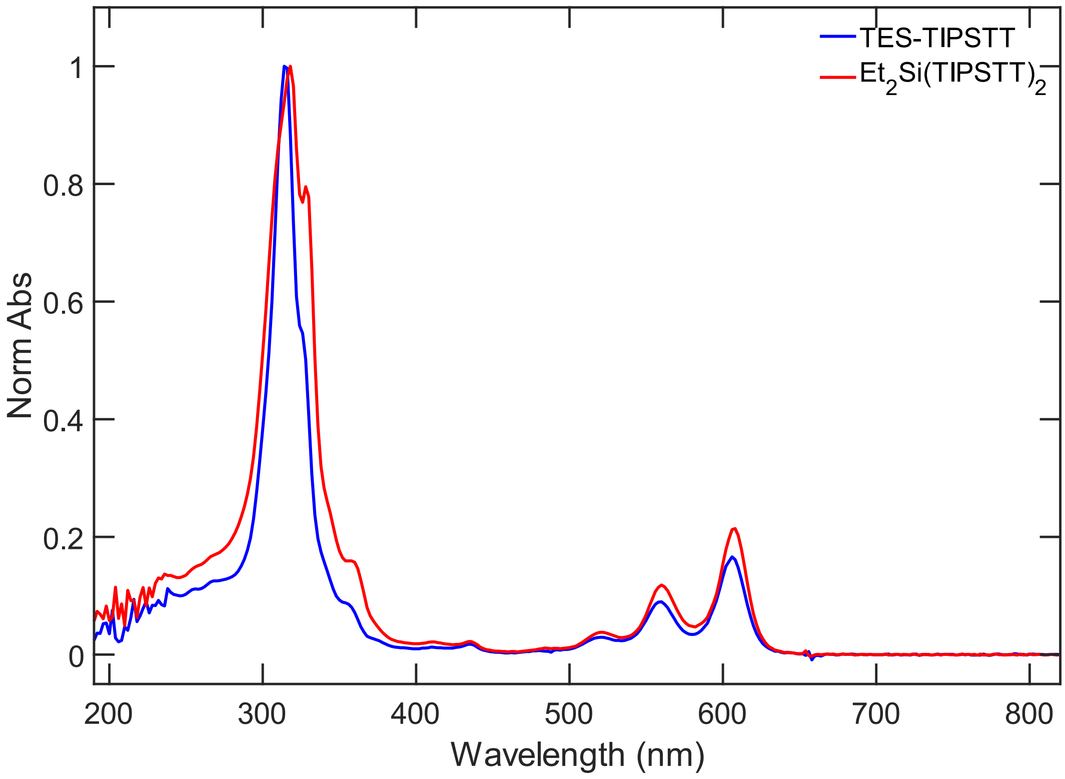

Figure S8. Normalized UVVis absorption spectra for **mono** and **dimer** measured in chloroform at room temperature.

3-2. Steady-state UVVis absorption and emission spectra of dimer in toluene and MeTHF

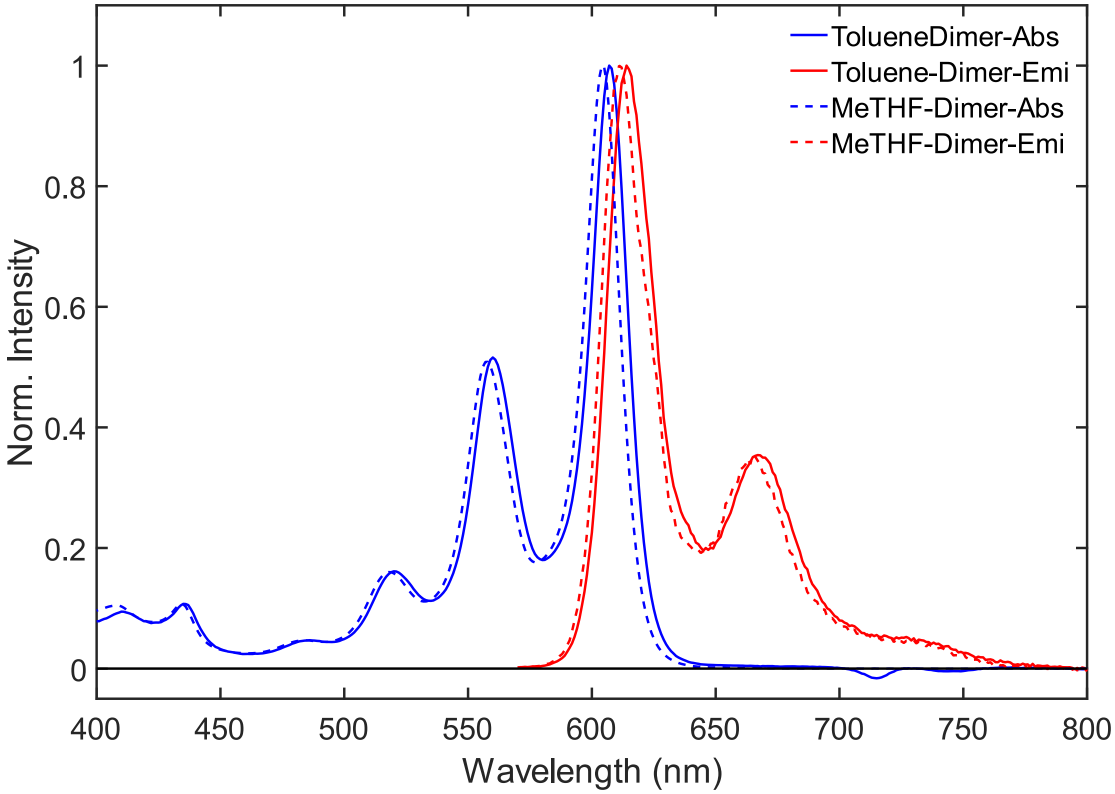

Figure S9. The emission and absorption spectra for **dimer** in Toluene and MeTHF at room temperature.

3-3. Steady-state UVVis absorption and emission spectra of mono and dimer in MeTHF

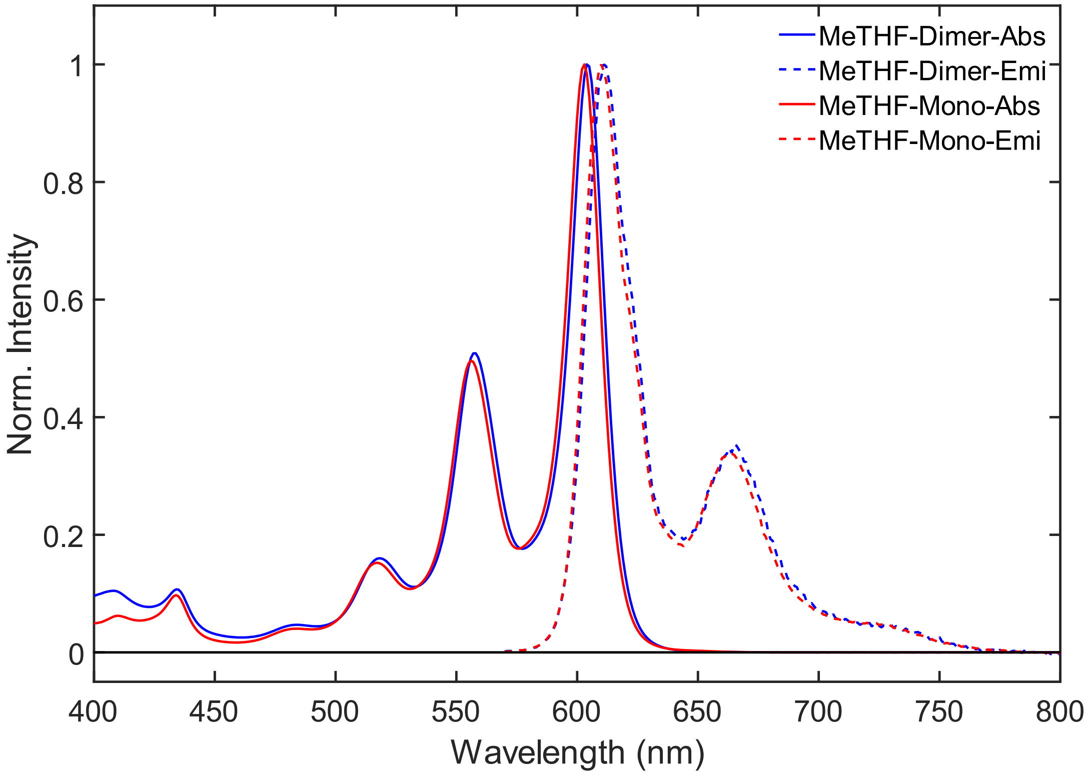

Figure S10. The emission and absorption spectra for **mono** and **dimer** in MeTHF at room temperature.

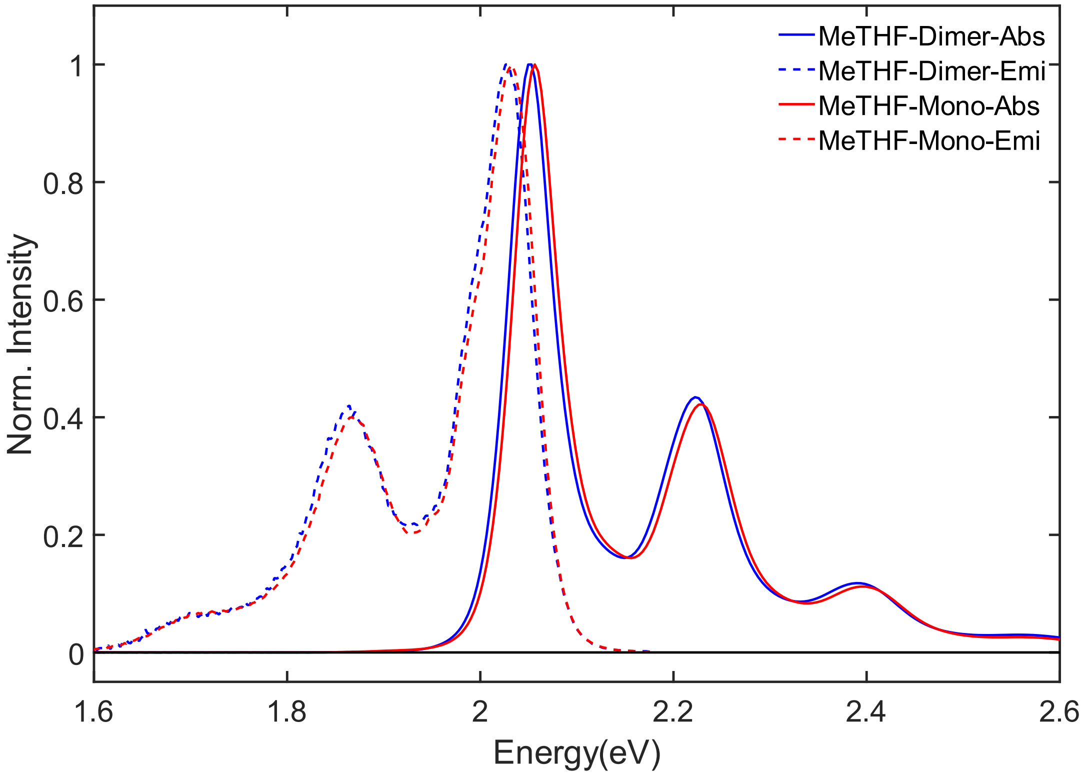

Figure S11. The emission and absorption spectra for **mono** and **dimer** in MeTHF at room temperature with x axis in unit of eV. The intensity is factored by Jacobian transformation.

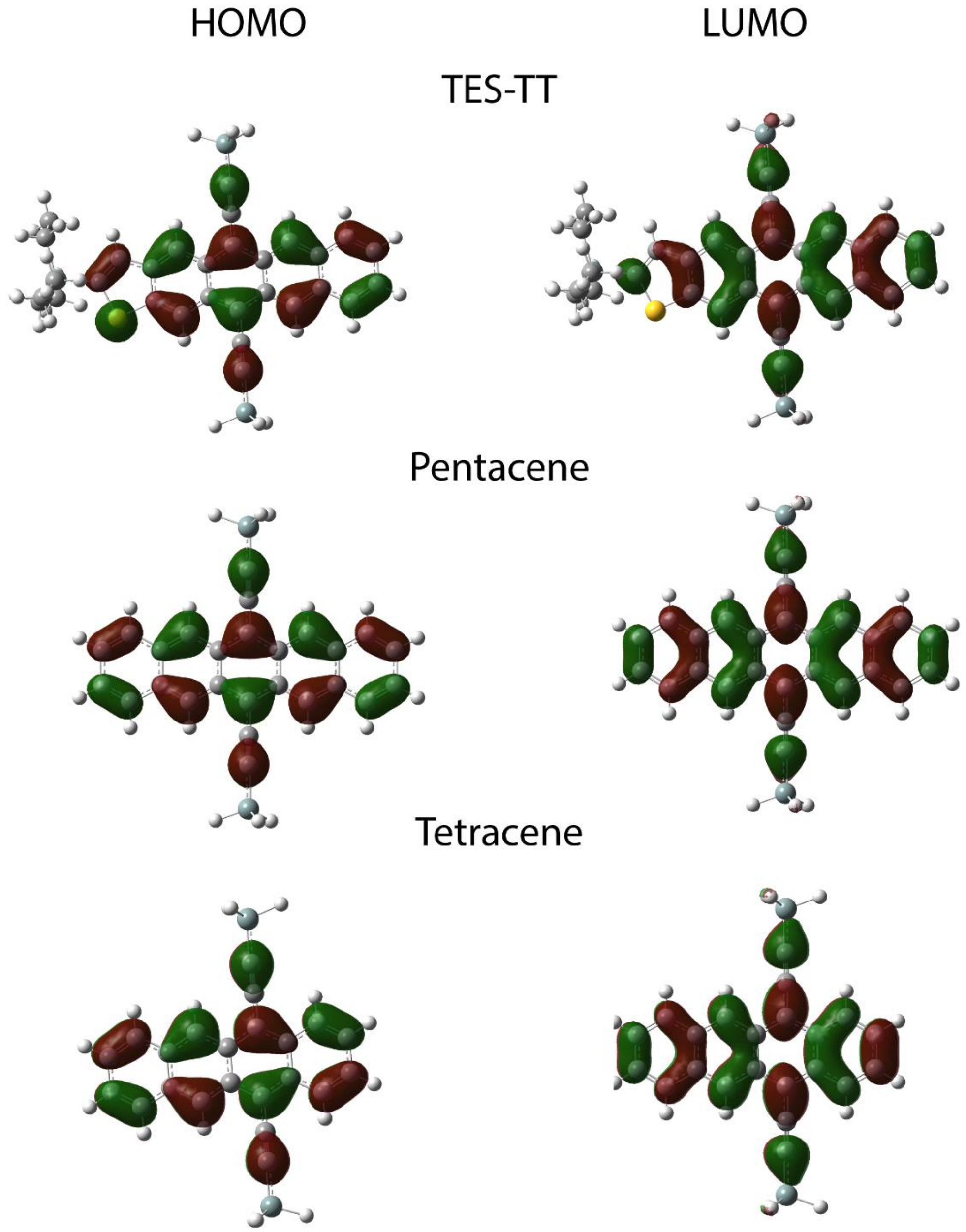

Figure S12. Molecular orbital surface for HOMO and LUMO of **mono**, pentacene and tetracene from TDDFT calculations with wb97xd/6-31g(d).

Table S1. Computed singlet and triplet vertical excitation energies for TIPS-Tetracene, TIPS-Thienotetracene and TIPS-Pentacene, and the S1/T1 energy ratios. All values in eV. Computed using ωB97XD/6-31G* in CHCl3 polarisable continuum. Triplet excitations calculated with the Tamm-Dancoff approximation.

	S_0-S_1	S_0-T_1	S_1/T_1
TIPS-Pentacene	1.86	0.88	2.11
TIPS-Thienotetracene	2.10	1.10	1.90
TIPS-Tetracene	2.34	1.29	1.82

4. Optimized geometries of $Et_2Si(SiH_3TT)_2$ for three conformers

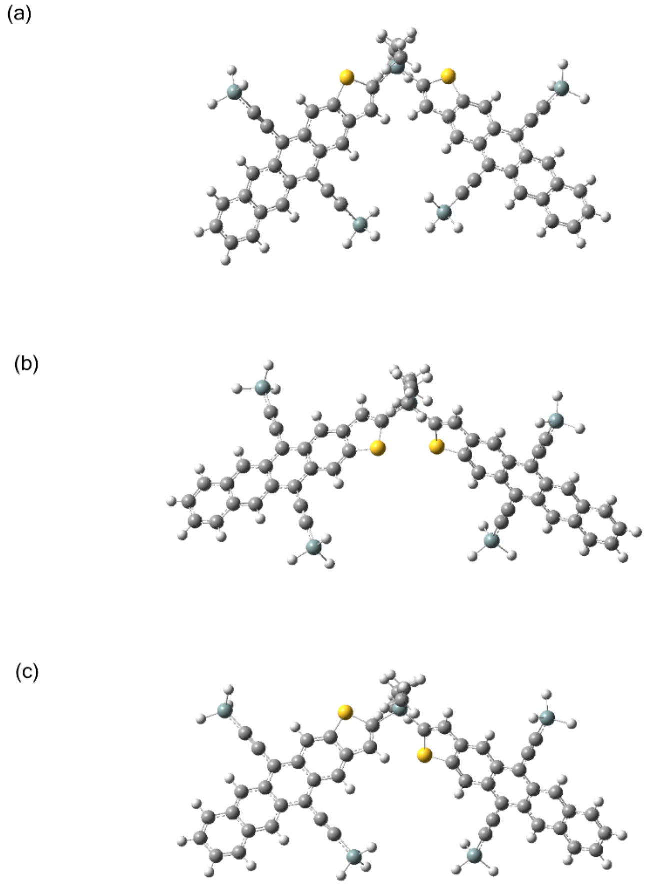

Figure S13. Ground state (S_0) geometries for the three conformers of $Et_2Si(SiH_3TT)_2$: (a) $Et_2Si(SiH_3TT)_2$-s (b) $Et_2Si(SiH_3TT)_2$-a (c) $Et_2Si(SiH_3TT)_2$-ns.

Table S2. The relative energetics between the three isomers with respect to $Et_2Si(SiH_3TT)_2$-s, which has the lowest ground state energy calculated by Rwb97xd/6-31g(d).

Structure	Rel. energy (meV)
$Et_2Si(SiH_3TT)_2$-ns	17.23
$Et_2Si(SiH_3TT)_2$-s	0.00
$Et_2Si(SiH_3TT)_2$-a	56.83

5. TCSPC measurement - dimer

The TCSPC data were fit simultaneously at all wavelengths, to a shared time-dependent function with wavelength dependent amplitude, using a global fitting algorithm that was written in-house. The time-dependence is described by a sum of two exponentially modified Gaussian functions (the analytical convolution of a Gaussian rise with an exponential decay; this function is discussed in the supporting information of Cook 2017[10]). The exponentially modified Gaussian function is used to describe the signal rise and early component; the Gaussian parameters are fixed to the values found from a Gaussian fit of the IRF, and the early component time constant is fixed to the value obtained from ultrafast TA measurements (107 ps). The value obtained for the longer time constant, 18.9 ± 0.02 ns.

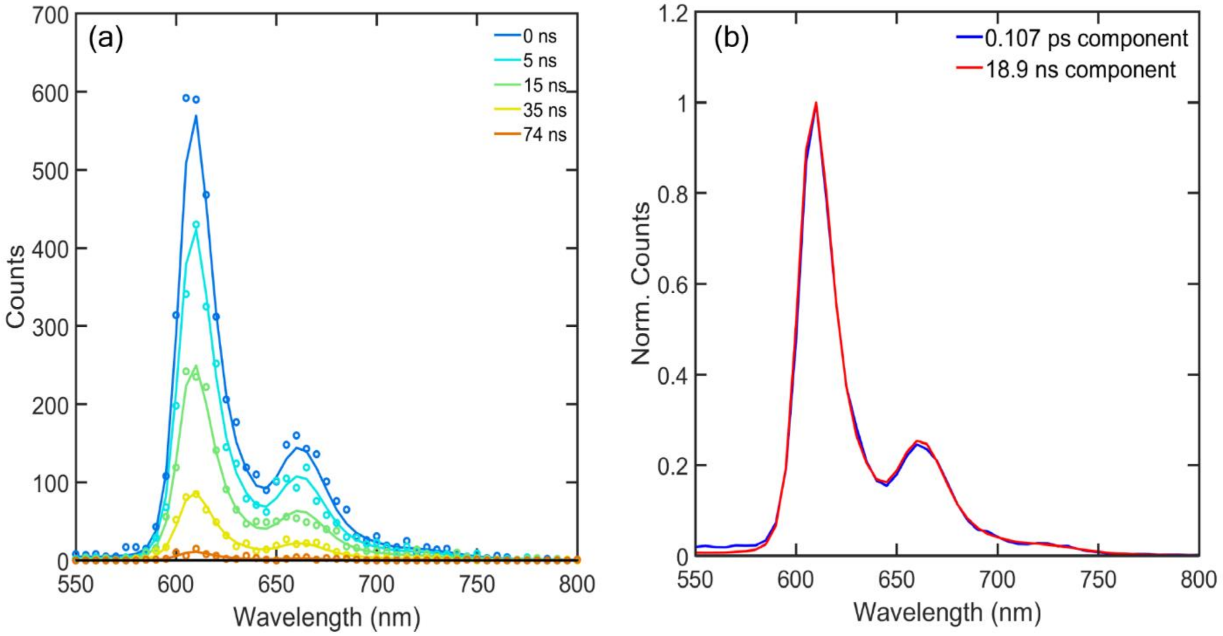

Figure S14. (a) Selected spectral slices for TCSPC measurement on **dimer** in MeTHF at room temperature. (b) Normalized species associated spectra for the two components of the decay lifetimes: 0.107 ps and 18.9 ns, extracted from global fit with exponentially modified gaussian function.

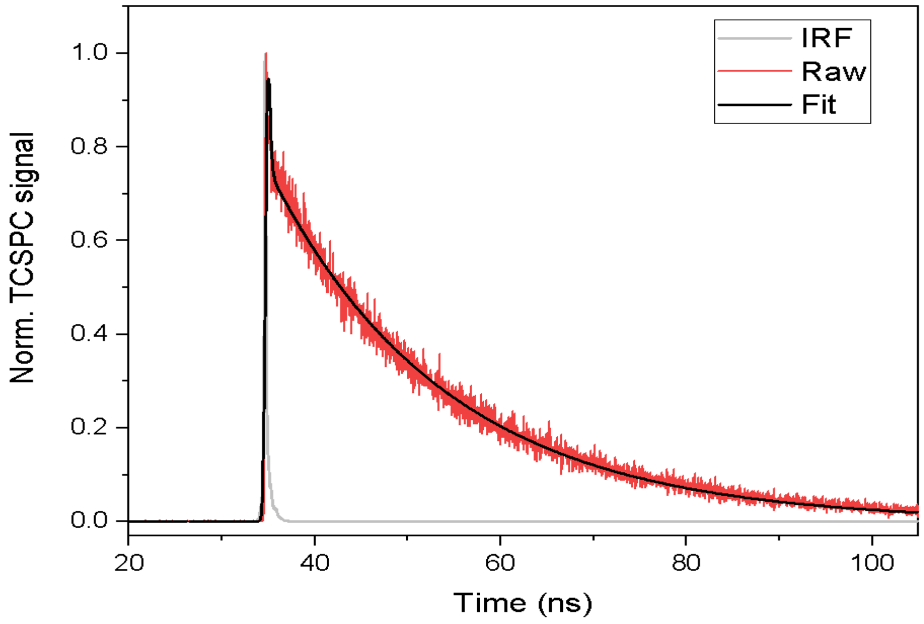

Figure S15. TCSPC time trace at 610 nm for **dimer** in MeTHF at room temperature. Normalized IRF (instrument response function) in grey solid line, raw data in red solid line and the fit with exponentially modified gaussian function. The early sharp decay lifetime is determined by fsTA, 107.2 ps. The later decay lifetime is 18.9 ns.

6. Ultrafast TA fitting - dimer

6-1. A→B→0 model

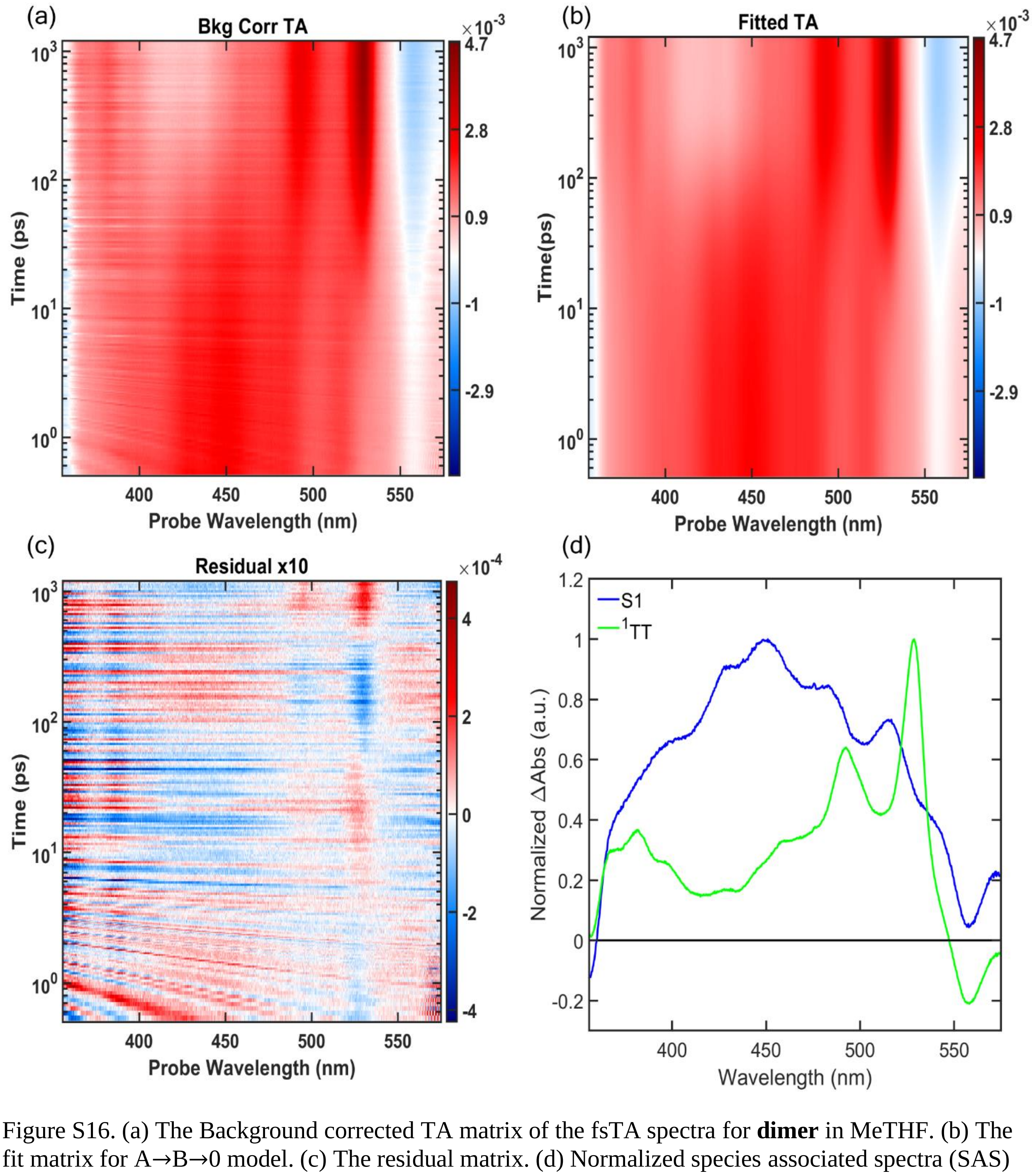

Figure S16. (a) The Background corrected TA matrix of the fsTA spectra for **dimer** in MeTHF. (b) The fit matrix for A→B→0 model. (c) The residual matrix. (d) Normalized species associated spectra (SAS) from the A→B→0 fitting model.

6-2. A→B→C→0 model

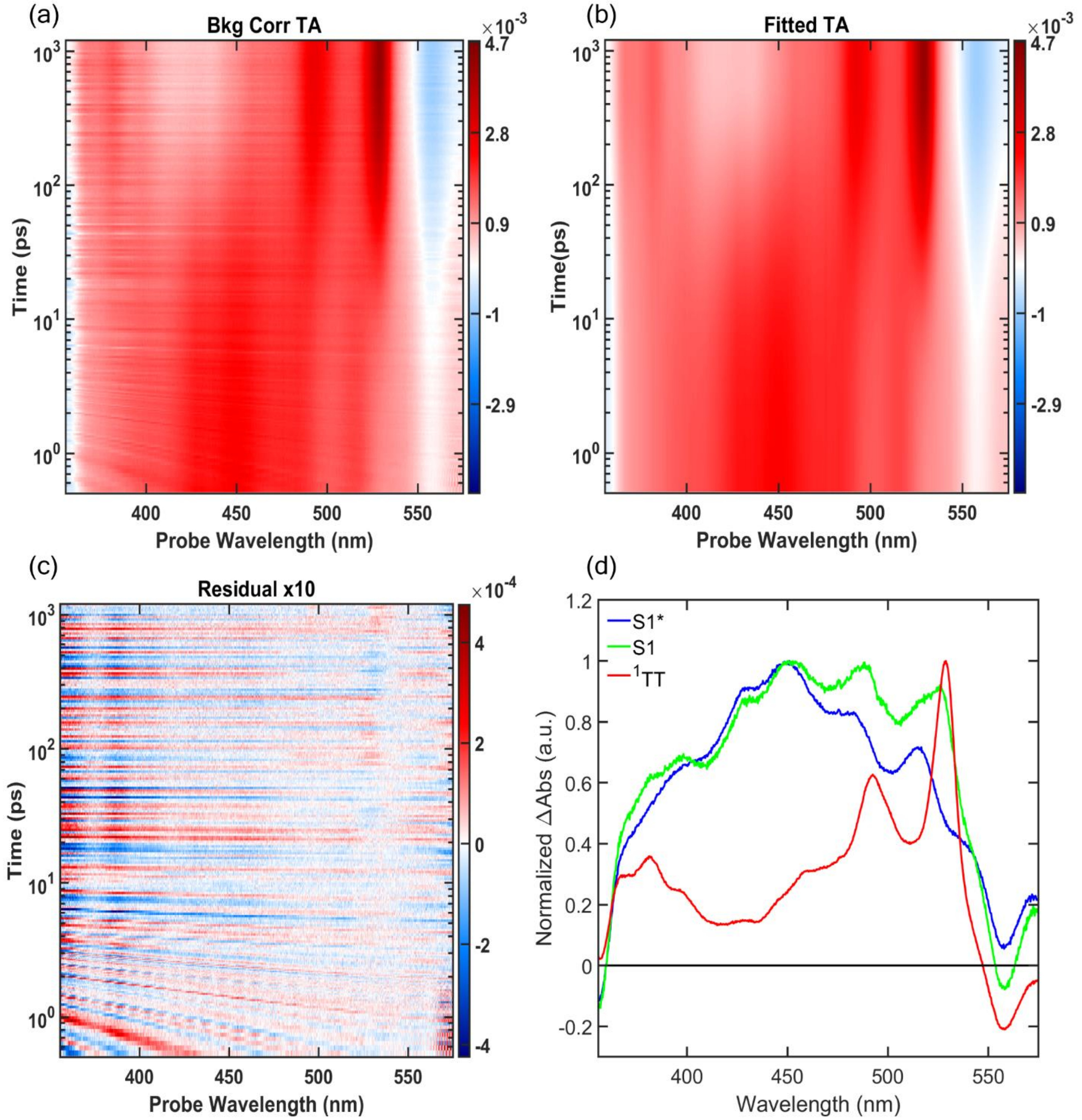

Figure S17. (a) The Background corrected TA matrix of the fsTA spectra for **dimer** in MeTHF. (b) The fit matrix for A→B→C→0 model. (c) The residual matrix. (d) Normalized species associated spectra (SAS) from the A→B→C→0 fitting model.

6-3. A (stretched)→B→0 model

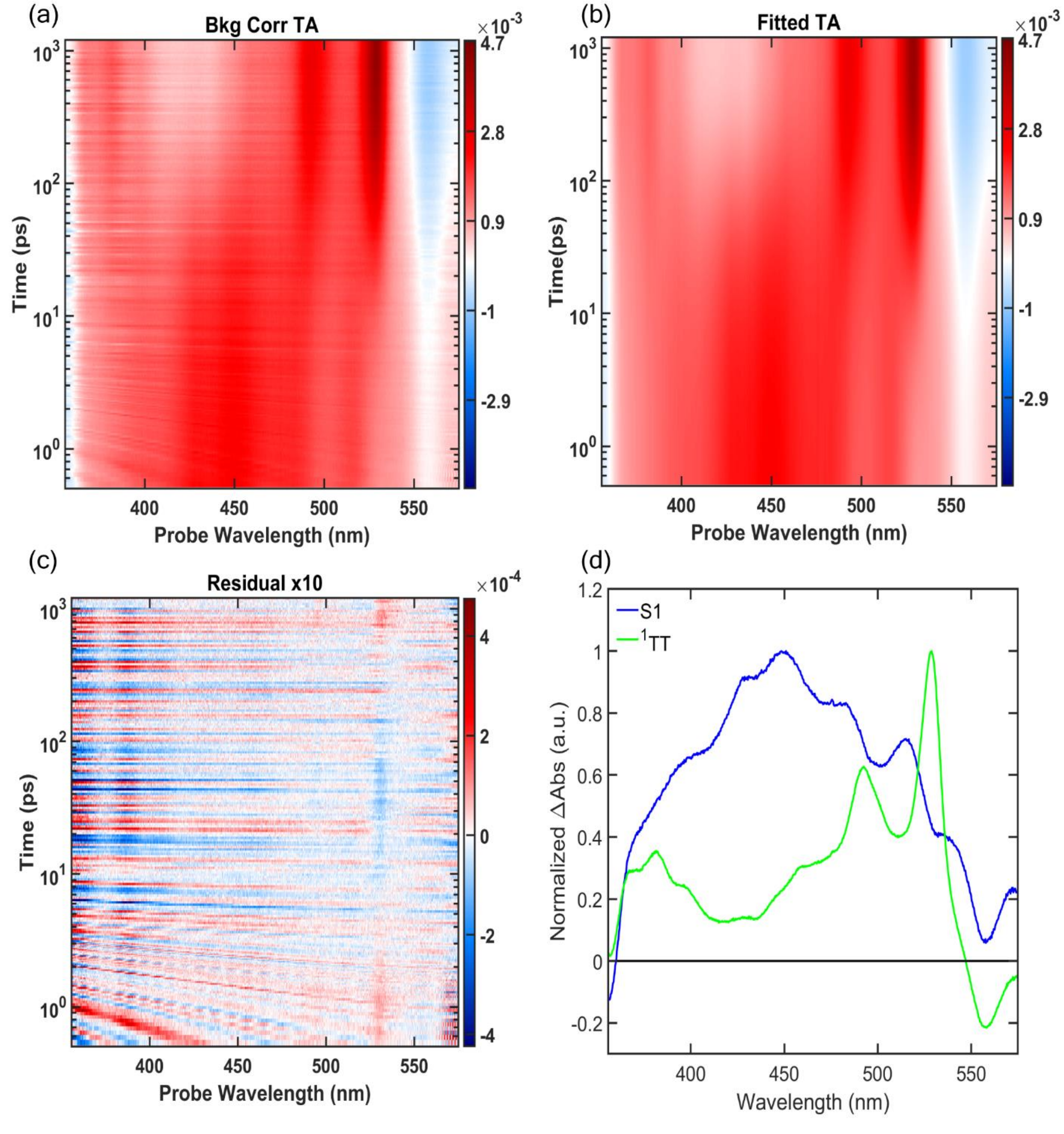

Figure S18. (a) The Background corrected TA matrix of the fsTA spectra for **dimer** in MeTHF. (b) The fit matrix for A (stretched)→B→0 model. (c) The residual matrix. (d) Normalized species associated spectra (SAS) from the A(stretched)→B→0 fitting model.

Table S3. Extracted S_1 lifetimes from model: A→B, A→B→C, and A (stretched) →B, where S_1 corresponds to A, B, and A (stretched), respectively.

Model	Lifetime of S_1 (ps)
A→B	94.3 ± 0.5
A→B->C	155.1 ± 2.1
A (stretched) →B	107.2 ± 0.7

7. Solvent dependent singlet fission of dimer via fs-TA

7-1. dimer in toluene

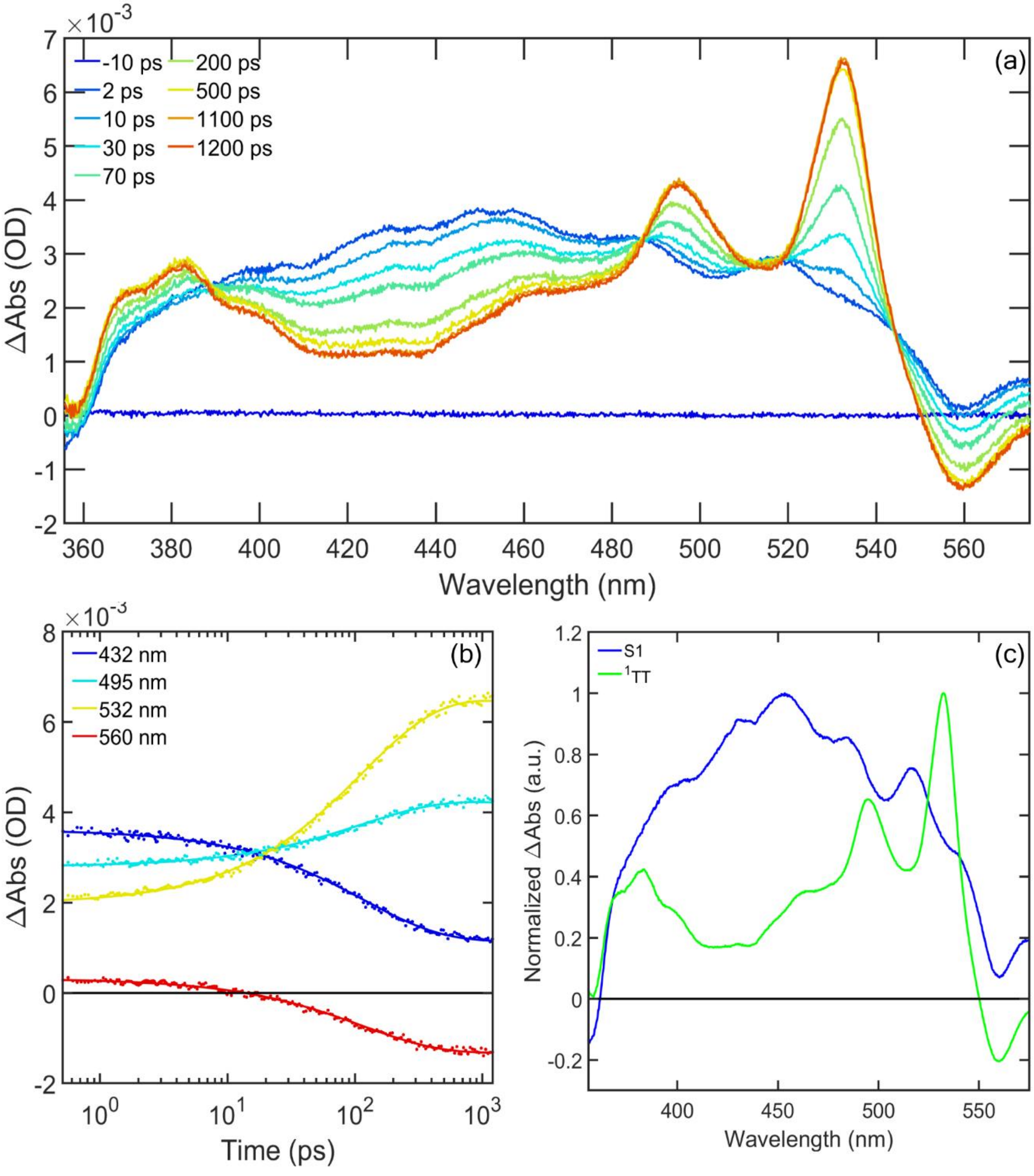

Figure S19. (a) fs-TA spectra of **dimer** in toluene at room temperature. The excitation wavelength is at a center wavelength of 604 nm which corresponds to $S_1 \leftarrow S_0$, 0-0 transition. (b) The selected single wavelength decay time traces (dots) for $Et_2Si(TIPSTT)_2$ with the A (stretched)→B→0 fitting model (solid lines) (c) The normalized species associated spectra for the S_1 and ^1TT.

7-2. dimer in benzonitrile

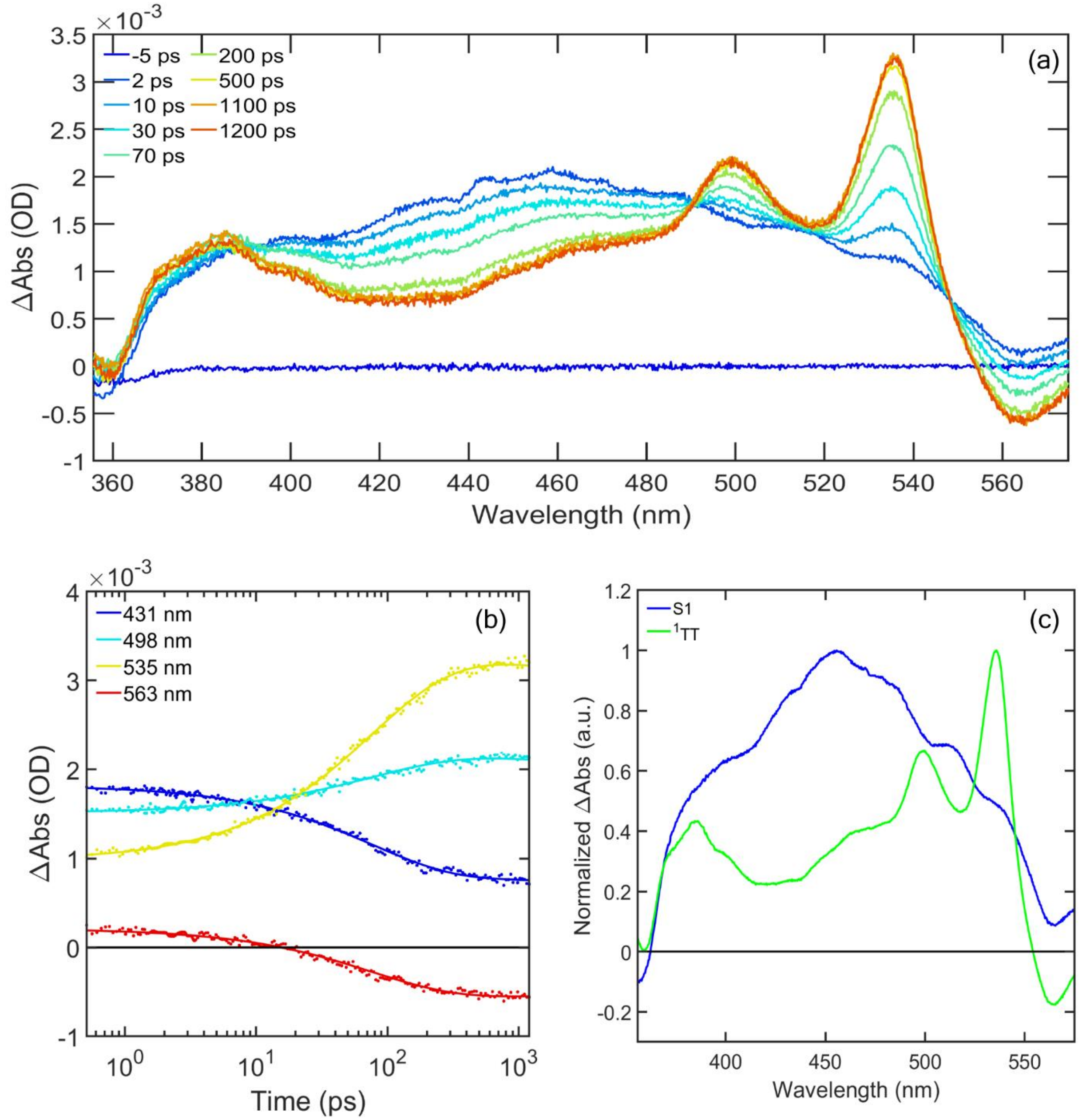

Figure S20. (a) fs-TA spectra of **dimer** in benzonitrile at room temperature. The excitation wavelength is at a center wavelength of 604 nm which corresponds to $S_1 \leftarrow S_0$, 0-0 transition. (b) The selected single wavelength decay time traces (dots) for **dimer** with the A (stretched)→B→0 fitting model (solid lines) (c) The normalized species associated spectra for the S1 and ^{1}TT.

8. mono fsTA & nsTA & TCSPC measurements

8-1. mono fsTA

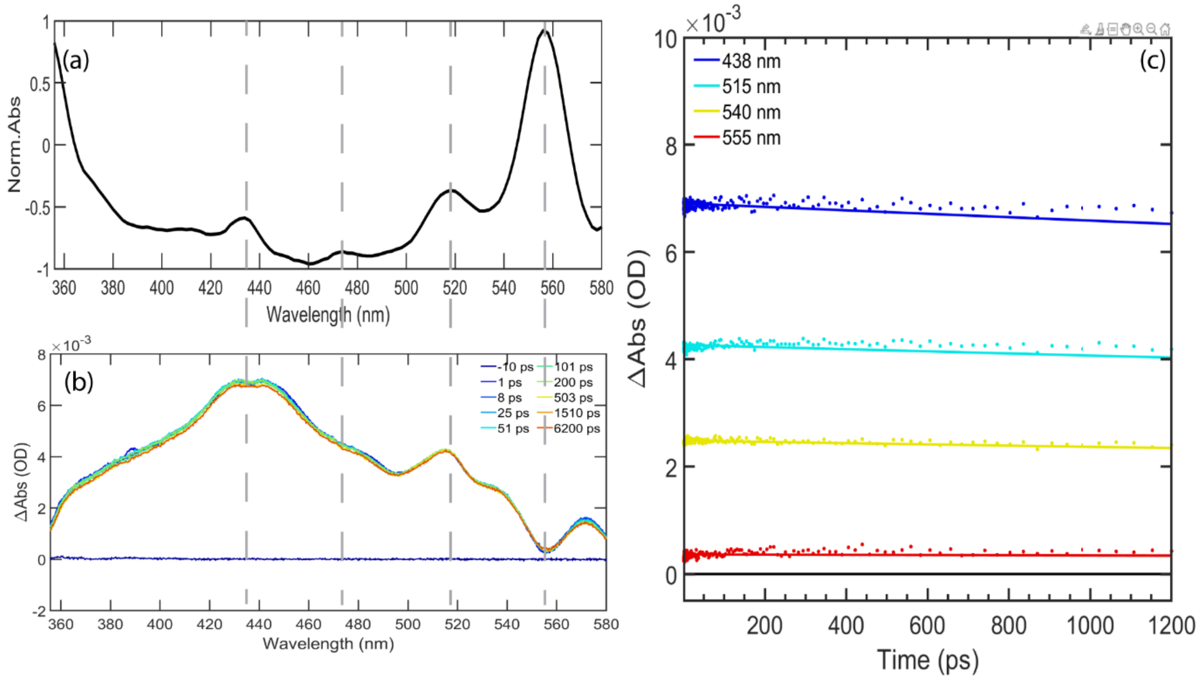

Figure S21. (a) Normalized absorption spectrum for **mono** in MeTHF at room temperature. (b) fsTA spectra of **mono** in MeTHF at room temperature. (c) The selected single wavelength decay time traces (dots) from (b) with a single exponential decay fitting model (solid lines).

8-2. mono nsTA and TCSPC

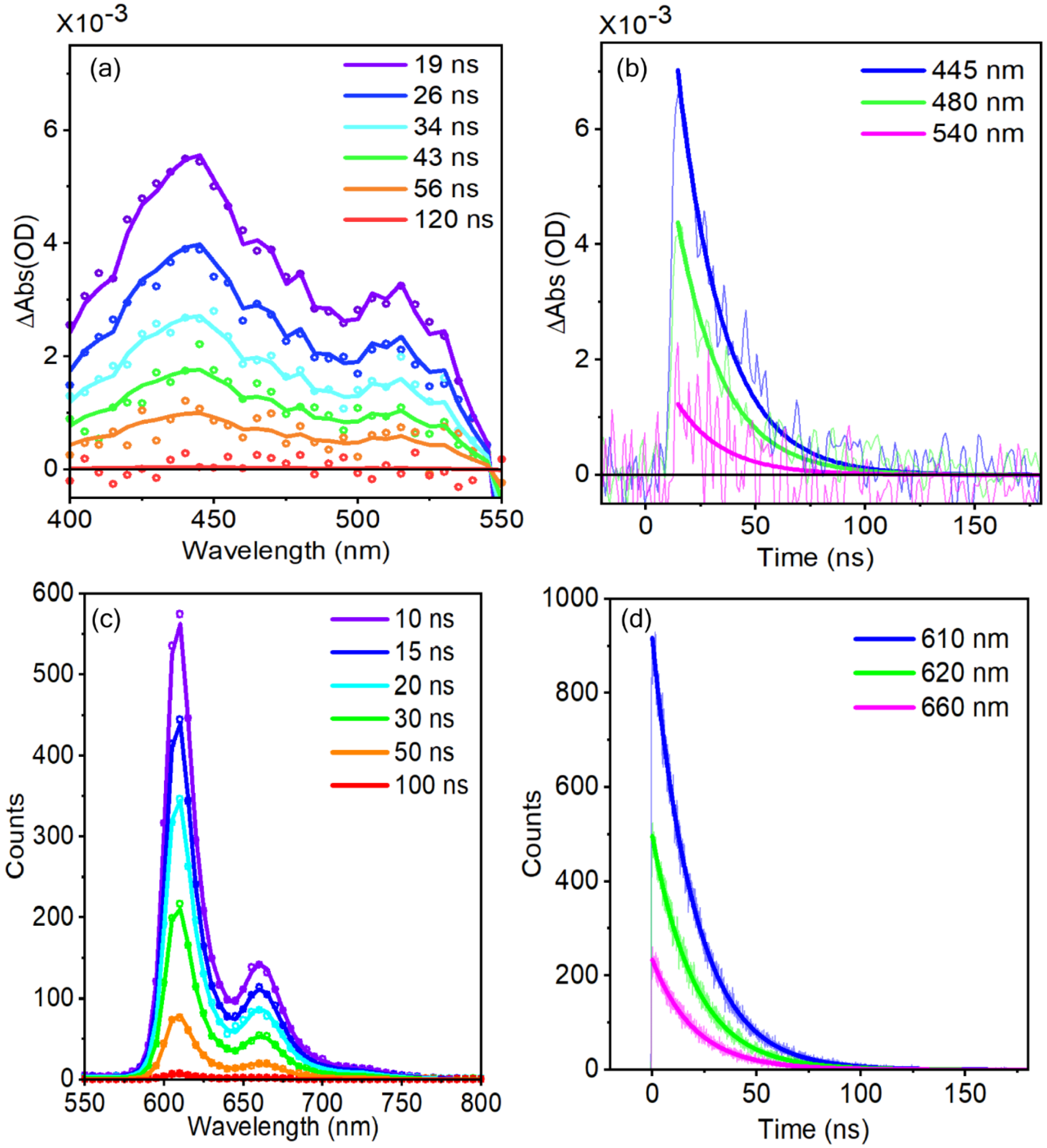

Figure S22. (a) nsTA spectra of **mono** in MeTHF at room temperature. Opened circles are from raw data. Solid lines are from global fit. (b) The selected single wavelength decay time traces (transparent lines) from (a) with a single exponential decay fitting model (bold lines). (c) TCSPC spectra of **mono** in MeTHF at room temperature. Opened circles are from raw data. Solid lines are from global fit. (d) The selected single wavelength decay time traces (transparent lines) from (c) with a single exponential decay fitting model (bold lines).

9. dimer temperature dependent nsTA

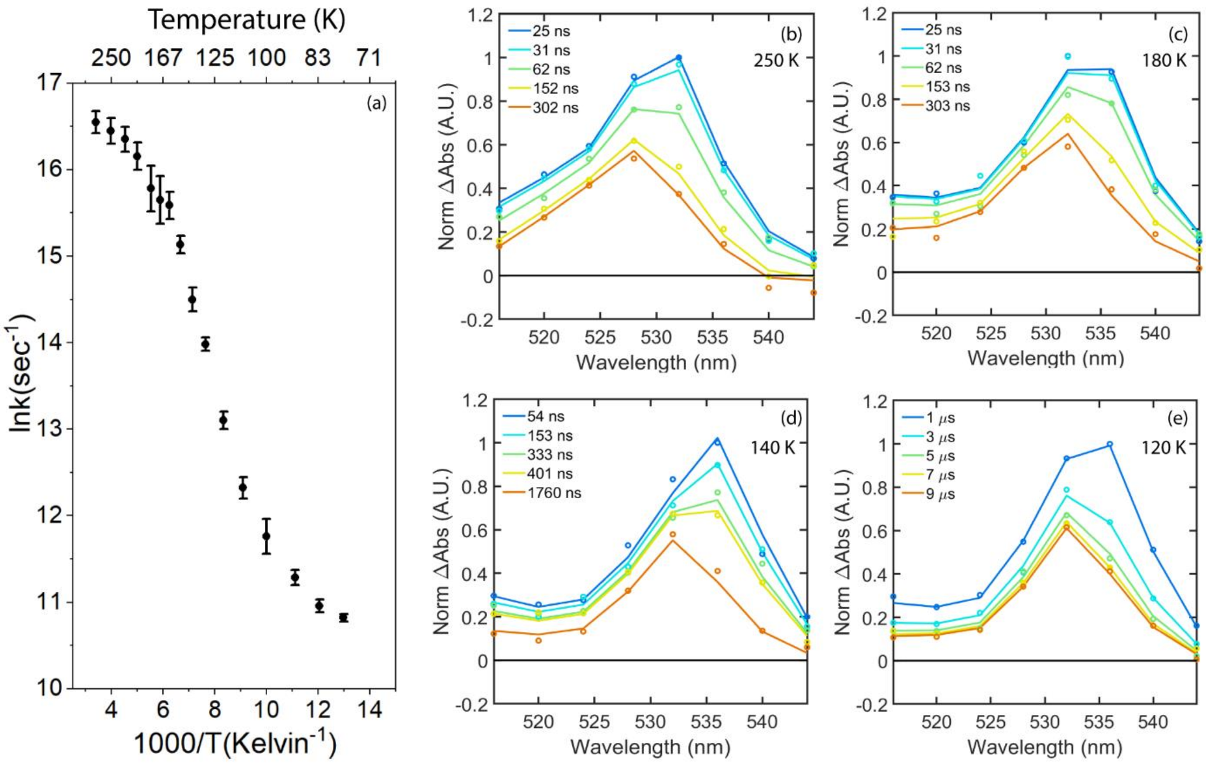

Figure S23. (a) Arrhenius plot for ^{n}TT decay rate coefficient (k_{dec}) in MeTHF. (b-e) ^{n}TT decay spectra from 516 nm to 544 nm at 250 K,180 K, 140 K and 120 K. Opened circles are from raw data. Solid lines are from global fit.

10.Species associated spectra (SAS)

10-1. SAS for nsTA at room temperature

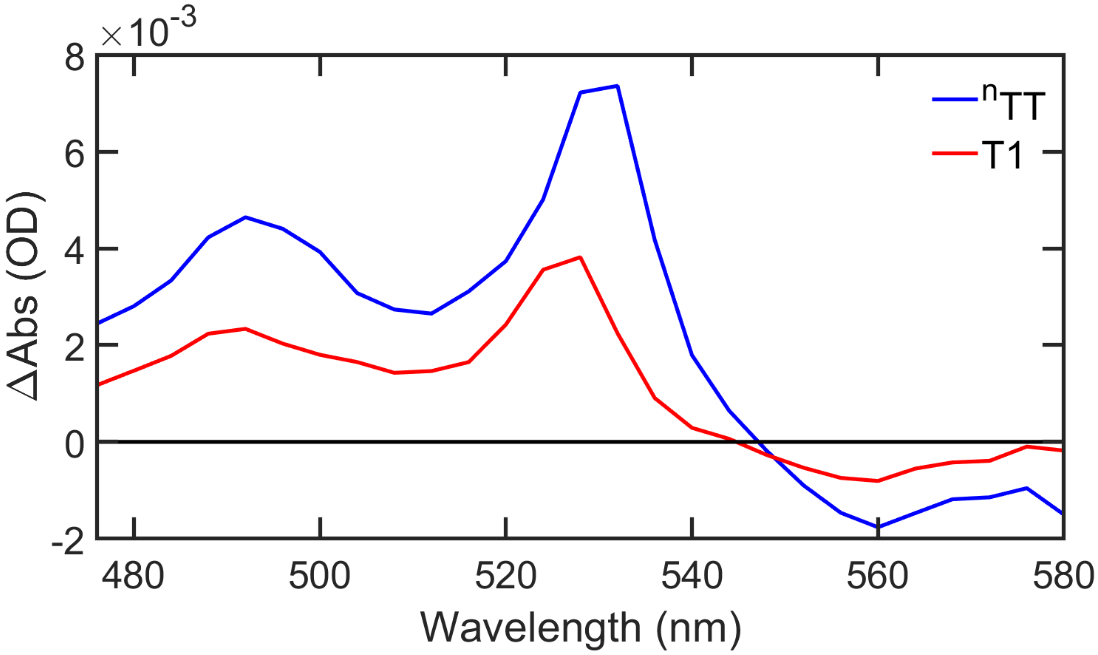

Figure S24. The species associated spectra for the two species, which are ^{n}TT and T_1 with lifetime of 62.5 ns and 26.6 μs, respectively, for **dimer** in MeTHF at room temperature from nsTA.

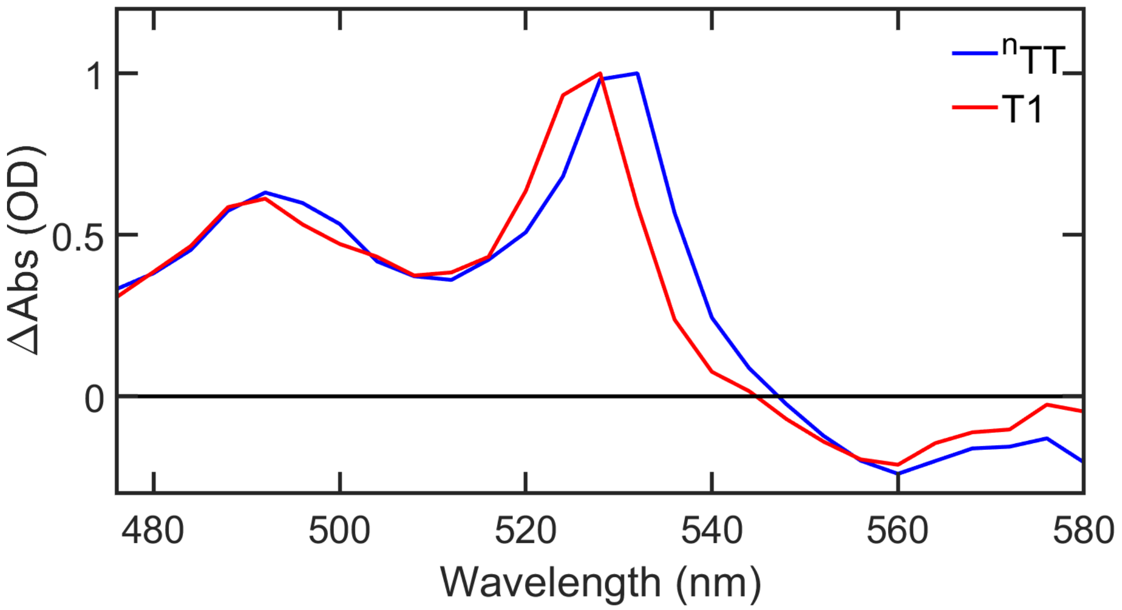

Figure S25. The normalized species associated spectra for the two species, which are ^{n}TT and T_1 with lifetime of 62.5 ns and 26.6 μs, respectively, for **dimer** in 2-MeTHF at room temperature from nsTA.

10-2. SAS for nsTA at 77 K

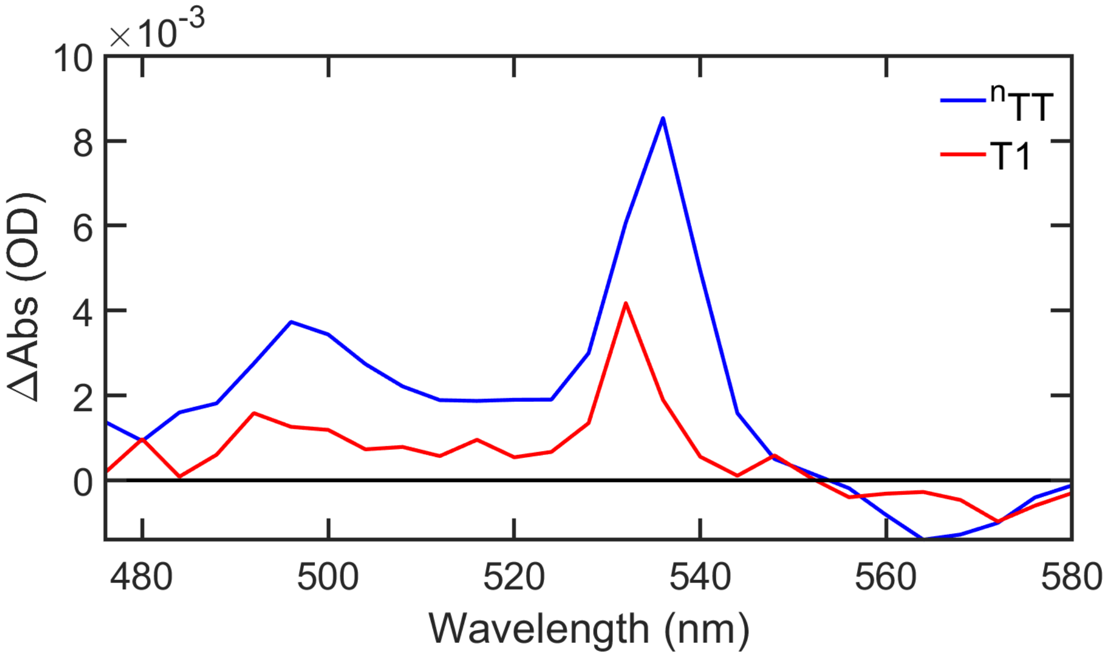

Figure S26. The species associated spectra for the two species, which are ^{n}TT and T_1 with lifetime of 16.9 μs and 58.1 μs, respectively, for **dimer** in MeTHF at 77K from nsTA.

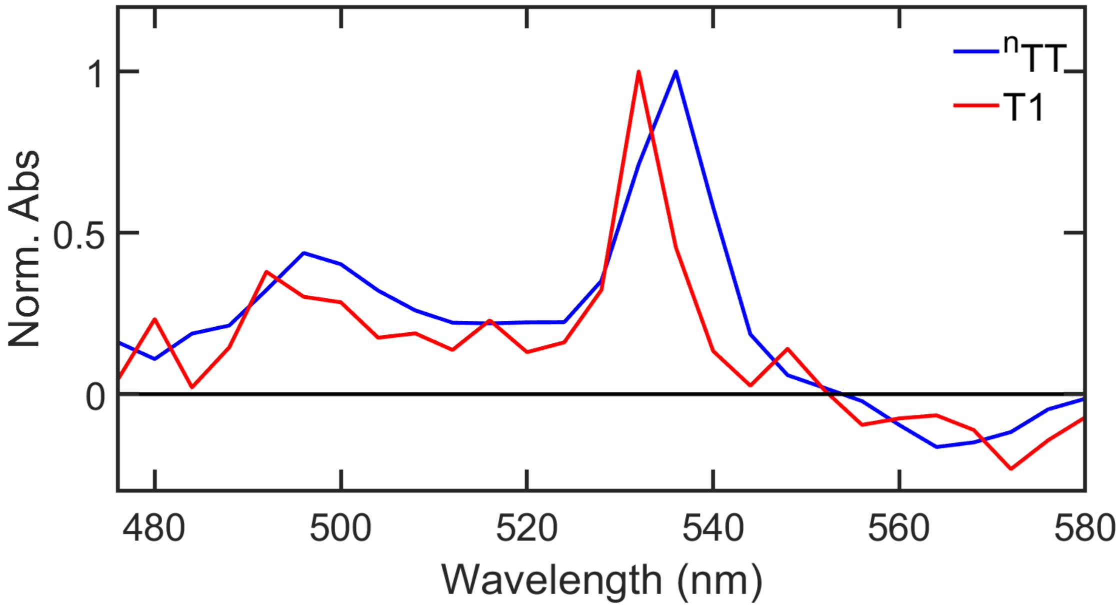

Figure S27. The normalized species associated spectra for the two species, which are ^{n}TT and T_1 with lifetime of 16.9 μs and 58.1 μs, respectively, for **dimer** in MeTHF at 77K from nsTA.

11. Triplet sensitization of mono

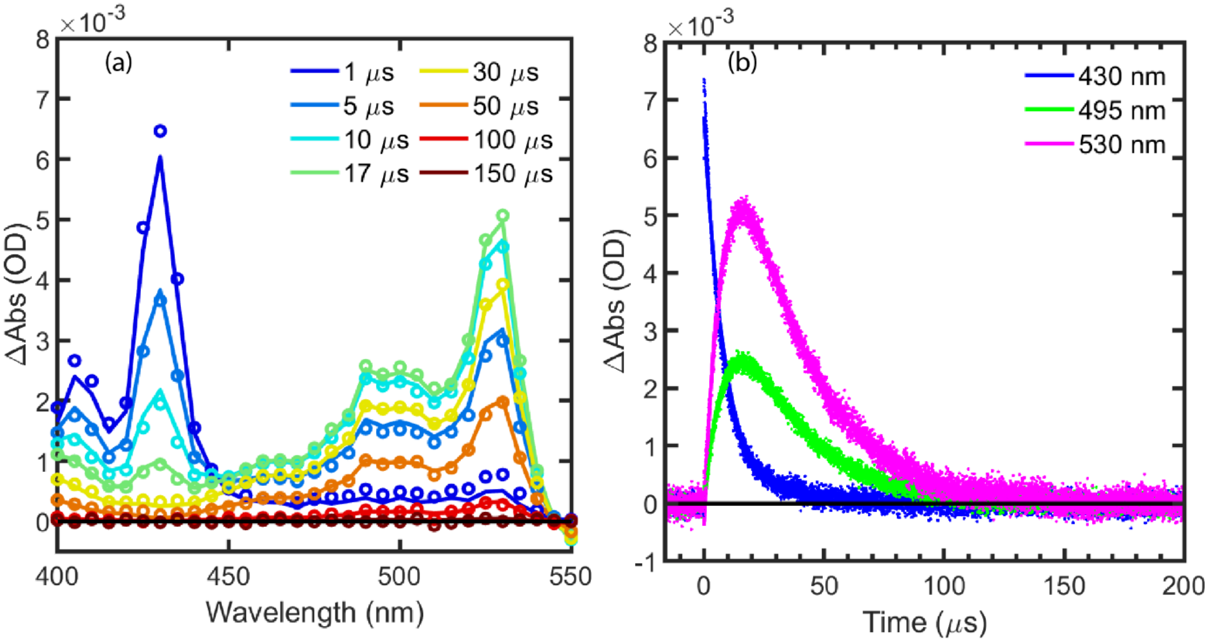

Figure S28. (a) nsTA spectra for **mono** sensitization with anthracene in toluene at 298K. Opened circles are from raw data. Solid lines are from global fit. (b) The selected single wavelength decay time traces (transparent) from (a) with bold solid lines from the global fit. The lifetime of the sensitized triplet is 27.7±0.1 μs.

12. trEPR and pulsed EPR measurement on dimer at 10K

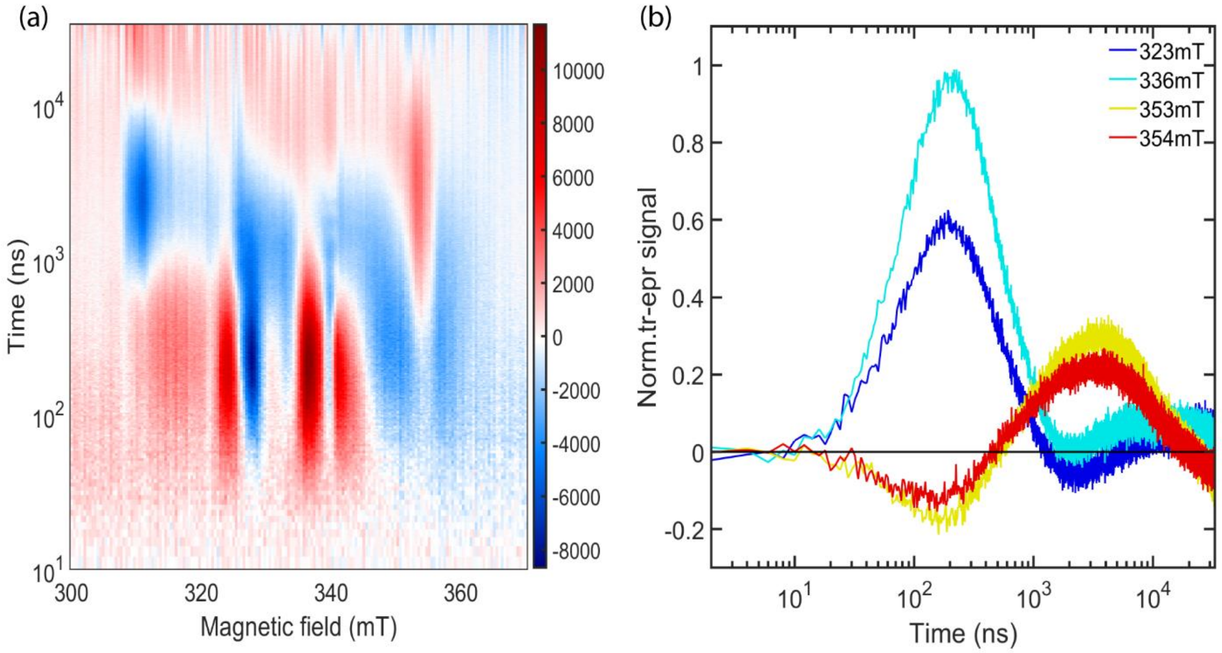

Figure S29. (a) trEPR matrix for **dimer** in MeTHF at 10 K. (b) The time traces at the selected magnetic fields.

13. Emission quantum yield measurement

Emission quantum yield is defined as the ratio of the number of photons emitted to the number of photons absorbed. We measure and calculate emission quantum yield for **dimer** and **mono** by comparing them with oxazine 720 in methanol, which has an emission quantum yield of 0.63. Take one of **mono**/MeTHF emission quantum yield measurement as an example. In Figure S30, we have steady-state absorption spectra for **mono**/MeTHF and oxazine/methanol. In addition, in Figure S31, we have steady-state emission spectra for **mono**/MeTHF and oxazine/methanol. Now we have everything needed for emission quantum yield calculation by plugging the experimental values into the equation: $\Phi = \Phi_R \frac{I}{I_R} \frac{OD_R}{OD} \frac{n^2}{n_R^2}$, where the subscript, R, means reference compound, oxazine/methanol, Φ is the emission quantum yield, I is the spectral integration of the emission spectra, OD is the number of photons absorbed,w hich is proportional to $(1 - 10^{-A})$, where A is the absorbance at the excitation frequency, and n is the refractive index. The error bar for the emission quantum yield measurement is ~ $\pm 10\%$. Table S4 shows the emission quantum yield values for mono/MeTHF and dimer/MeTHF from three independent measurements.

Table S4. Emission quantum yield for **mono**/MeTHF and **dimer**/MeTHF for the three independent measurements.

	Emission Quantum yield
**mono**/MeTHF	1.02 ±0.11
**dimer**/MeTHF	0.12 ± 0.02

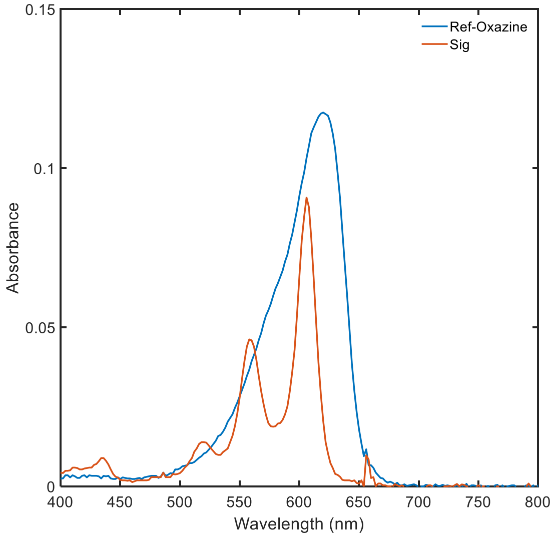

Figure S30. Absorption spectra for Oxazine/Methanol (Ref-Oxazine, blue) and **mono**/MeTHF (Sig, red).

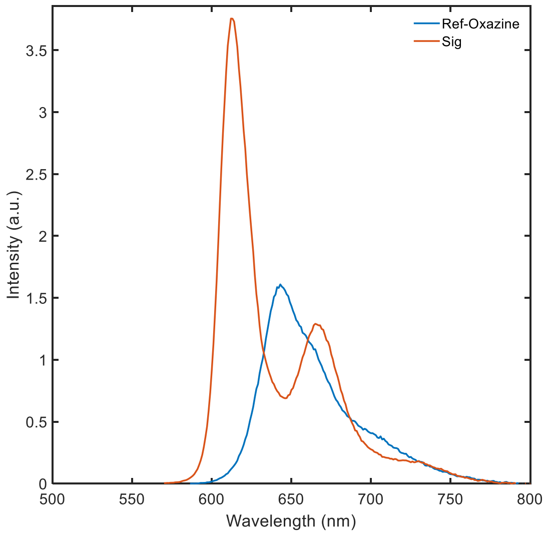

Figure S31. Emission spectra for Oxazine/Methanol (Ref-Oxazine, blue) and **mono**/MeTHF (Sig, red).

Reference

1. Tang, M. L., Reichardt, A. D., Siegrist, T., Mannsfeld, S. C. B. & Bao, Z. Trialkylsilylethynyl-Functionalized Tetraceno[2,3- *b*]thiophene and Anthra[2,3- *b*]thiophene Organic Transistors. *Chem. Mater.* **20**, 4669–4676 (2008).
2. Rugg, B. K. *et al.* Triplet-pair spin signatures from macroscopically aligned heteroacenes in an oriented single crystal. *Proc. Natl. Acad. Sci. U.S.A.* **119**, e2201879119 (2022).
3. Krause, L., Herbst-Irmer, R., Sheldrick, G. M. & Stalke, D. Comparison of silver and molybdenum microfocus X-ray sources for single-crystal structure determination. *J Appl Crystallogr* **48**, 3–10 (2015).
4. Sheldrick, G. M. Crystal structure refinement with *SHELXL*. *Acta Crystallogr C Struct Chem* **71**, 3–8 (2015).
5. Parkin, S. Expansion of scalar validation criteria to three dimensions: the *R* tensor. *Acta Crystallogr A Found Crystallogr* **56**, 157–162 (2000).
6. Spek, A. L. Structure validation in chemical crystallography. *Acta Crystallogr D Biol Crystallogr* **65**, 148–155 (2009).
7. *International tables for crystallography. C: Mathematical, physical and chemical tables / ed. by E. Prince*. (Kluwer Academic, 2004).
8. Fudickar, W. & Linker, T. Why Triple Bonds Protect Acenes from Oxidation and Decomposition. *J. Am. Chem. Soc.* **134**, 15071–15082 (2012).
9. Dill, R. D., Smyser, K. E., Rugg, B. K., Damrauer, N. H. & Eaves, J. D. Entangled spin-polarized excitons from singlet fission in a rigid dimer. *Nat Commun* **14**, 1180 (2023).

10. Cook, J. D., Carey, T. J., Arias, D. H., Johnson, J. C. & Damrauer, N. H. Solvent-Controlled Branching of Localized versus Delocalized Singlet Exciton States and Equilibration with Charge Transfer in a Structurally Well-Defined Tetracene Dimer. *J. Phys. Chem. A* **121**, 9229–9242 (2017).
